# The attraction effect in perceptual decision-making: a case of dominance asymmetry

**DOI:** 10.3389/fpsyg.2025.1661748

**Published:** 2025-12-18

**Authors:** Tapas Rath, Narayanan Srinivasan, Nisheeth Srivastava

**Affiliations:** Department of Cognitive Science, Indian Institute of Technology Kanpur, Kanpur, India

**Keywords:** asymmetric dominance, attraction effect, preference reversals, perceptual decoy, decision making

## Abstract

**Introduction:**

The attraction effect (AE), or asymmetric dominance effect, occurs when the presence of a clearly inferior decoy increases the choice of a target over a competitor. While robust in value-based domains, findings with perceptual stimuli have been inconsistent, with some studies even reporting reversals in triangular arrangements of stimuli.

**Methods:**

Across four experiments and a reanalysis of prior data, we investigated whether these inconsistencies are attributable to the presence or absence of genuine item-wise dominance asymmetry. We utilized novel star-shaped stimuli and traditional rectangle stimuli to test the dominance asymmetry.

**Results:**

Experiment 1 established that the star-shaped stimuli reliably produced strong target-decoy (TD) dominance over competitor-decoy (CD) dominance, whereas traditional rectangles showed weaker but still positive asymmetry. Experiment 2 provided the first robust demonstration of a positive AE with perceptual stimuli in a triangular layout using stars, while Experiment 3 showed that rectangle stimuli, when presented in a triangular layout, produced an aggregate null rather than a negative effect. Similarly, the reanalysis of data from a previous triplet experiment involving bars stimuli pointed toward a null effect. Experiment 4 again linked inconsistent findings from linear vs. triangular alignment of triplet rectangles to the presence of asymmetric dominance, while also demonstrating an interaction between the differential ease of comparison in pairs and presentation format.

**Discussion:**

Together, these results demonstrate that AE is a robust phenomenon that emerges whenever decoys create strong item-level dominance asymmetry. Apparent inconsistencies with perceptual stimuli reflect stimulus-specific dominance structures. This work clarifies the boundary conditions of the AE, reinforces its domain generality, and provides methodological guidance for future research on context effects in perceptual decision-making.

## 1 General introduction

The principle of *Independence of Irrelevant Alternatives* (IIA) is foundational in rational choice theory, stating that the relative odds of choosing between two alternatives should remain unchanged by the introduction or removal of additional alternatives (Luce, 1959):


$$\frac{P\left(i|C\right)}{P\left(j|C\right)}=\frac{P\left(i|C^{\prime }\right)}{P\left(j|C^{\prime }\right)}$$

for options *i, j* in sets *C* ⊂ *C*′.

The *regularity* axiom is a related, but weaker, requirement that adding an alternative cannot increase the probability of choosing any of the original options (Luce, 1977):


$$P\left(i|C^{\prime }\right)\leq P\left(i|C\right)$$

for any *i* in the original set.

Tversky's work in the 1970s empirically demonstrated violations of IIA—the “similarity effect” showed that new, similar alternatives could alter relative choice odds between original items, challenging the basic premises of deterministic choice theory (Tversky, 1972). In response to these findings, Luce observed that regularity was the only rational choice axiom yet unviolated by data (Luce, 1977). This status changed following the landmark study by Huber, Payne, and Puto (Huber et al., 1982), who showed that the *attraction effect* violates regularity: the addition of a decoy can *increase* the choice probability of an existing option. It is now recognized that regularity is a necessary but not sufficient condition for IIA: any violation of regularity implies a violation of IIA, but IIA can be violated without a corresponding violation of regularity.

The attraction effect (AE) or asymmetric dominance effect is a widely studied choice bias in decision-making where the presence of a third option (the “decoy”: D) influences decision-makers to prefer one of the original options (the “target”: T) over the other (the “competitor”: C). The phenomenon is both practically and theoretically important. Practically, it serves as a behavioral nudge to influence consumers' choices (Devine et al., 2025). Theoretically, as we discussed, it demonstrates a violation of the assumption in rational choice theory: regularity, and hence IIA.

Over the past forty years, researchers have documented the attraction effect across various decision-making contexts. For instance, choices under risk exhibit systematic shifts when a dominated alternative is introduced (Farmer et al., 2017), and category-based inference similarly shows sensitivity to contextual distractors (Trueblood, 2012; Choplin and Hummel, 2005). Preference for a target option also changes in typical consumer goods settings (Huber et al., 1982; Noguchi and Stewart, 2014), as well as during sequential motor planning tasks (Farmer et al., 2017). Beyond studies with adult humans, developmental and comparative studies reveal that this context-dependent shift occurs in young children (Zhen and Yu, 2016), domestic cats (Scarpi, 2011), nonhuman primates (Parrish et al., 2015), various avian species including hummingbirds (Bateson et al., 2003), amphibians such as frogs (Lea and Ryan, 2015), social insects like honeybees (Shafir et al., 2002), and even in slime molds (Latty and Beekman, 2011).

Studies by (Choplin and Hummel 2005) and (Trueblood et al. 2013), which demonstrated attraction effects even in the perceptual domain, became essential milestones in establishing the ubiquity of the effect and challenging the possibility of cardinal representations of value in the brain (Vlaev et al., 2011). Cardinal representations refer to models in which values are encoded on an absolute, context-independent scale, allowing comparisons to be made based on fixed neural metrics. The presence of attraction effects in both value-based and perceptual tasks indicates that the brain's encoding of magnitude is relative and context-dependent, rather than strictly cardinal. Demonstrating these effects in perceptual judgments–where attributes have clear physical units–provides strong evidence that context-dependent comparison processes are fundamental and not restricted to economic or subjective value domains.

Despite its wide documentation across species and domains, recent years have seen a growing number of studies reporting inconsistent, muted, or even reversed attraction effects (repulsion effect) (Frederick et al., 2014; Yang and Lynn, 2014). However, (Huber et al. 2014) identified several boundary conditions that can constrain the effect: strong prior preferences between core options, difficulty detecting dominance, individual variation in attribute weighting, and pronounced aversion to or preference for the decoy. They argued that studies failing to meet one or more of these criteria unsurprisingly reported inconsistent effects. Subsequent work has further highlighted such constraints. In the perceptual domain, (Spektor et al. 2018, 2022) found reversed effects when stimuli were arranged in a triangular configuration, leading some to question the domain generality of the attraction effect. Most notably, (Trendl et al. 2021) rigorously tested the attraction effect with naturalistic stimuli (movies), while satisfying all boundary conditions identified by (Huber et al. 2014). However, they observed a precisely zero effect, leading them to conclude that the attraction effect may be limited to stylized experimental stimuli.

We suggest that these recent failures may reflect the violation of an additional, previously overlooked boundary condition: the requirement that the decoy be truly asymmetrically dominated. We developed a novel class of perceptual stimuli that affords clear and unambiguous asymmetric dominance (explained below) and adopted a pairwise comparison framework commonly used in the choice literature (explained next) to test whether restoring this relational structure reinstates the standard attraction effect.

### 1.1 Asymmetric dominance of decoy

The attraction effect, also known as the asymmetric dominance effect, occurs when the presence of a clearly inferior decoy option increases the likelihood of choosing the target over the competitor (Huber et al., 1982). Central to this phenomenon is the notion of *asymmetric dominance*—the structural relationship between options in the choice set that differentiates targets, competitors, and decoys.

Historically, asymmetric dominance has been defined primarily in **attribute-based terms**, where a decoy is considered asymmetrically dominated if it is inferior to the target on *every* attribute but is partially dominated by (or equal to) the competitor on at least one attribute (Kaptein et al., 2016; Bhatia, 2013; Žofák, 2016; Helgadóttir, 2015). For example, a decoy may be dominated by the target in both price and quality but be better than or equal to the competitor in at least one attribute. This attribute-wise definition of asymmetric dominance has guided much of the experimental literature in designing decoys.

However, the original conceptualization by (Huber et al. 1982) adopts an **item-based** definition: an alternative is asymmetrically dominated if it is dominated by one option in the choice set but not by another. This perspective focuses on the overall dominance relationships between *whole options* rather than isolating attribute-level comparisons. Importantly, this item-based asymmetry is broader and encapsulates attribute-based definitions as a special case.

Formally, these definitions can be summarized as follows, where *D*_*x*_ and *D*_*y*_ denote decoy attribute values, *T*_*x*_ and *T*_*y*_ target attribute values, and *C*_*x*_ and *C*_*y*_ competitor attribute values:


$$\begin{array}{r} \text{Attribute-Based Asymmetric Dominance:}D_{x}\leq T_{x},\;D_{y}\leq T_{y},\\ D_{y}\leq C_{y},\;D_{x}>C_{x} \end{array}$$ (1)

$$\begin{array}{r} \text{Item-Based Asymmetric Dominance:}P\left(T|TD\right)\gg 0.5,\\ P\left(C|CD\right)\approx 0.5 \end{array}$$ (2)

where *P*(*X*∣*XY*) is the probability of choosing option *X* from binary pair *XY*, computable as choice frequencies.

It should be noted, however, that the above formalizations concern only structural definitions of asymmetry reflected in behavior, without any commitment to the underlying cognitive process. The distinction is important because it separates structural boundary conditions about dominance asymmetry from assumptions about the cognitive comparison process. Our approach adopts the item-based definition of asymmetry to remain as general as possible and focuses on measurable choice behavior without committing to specific cognitive mechanisms (Banerjee et al., 2024b).

**Figure 1:**
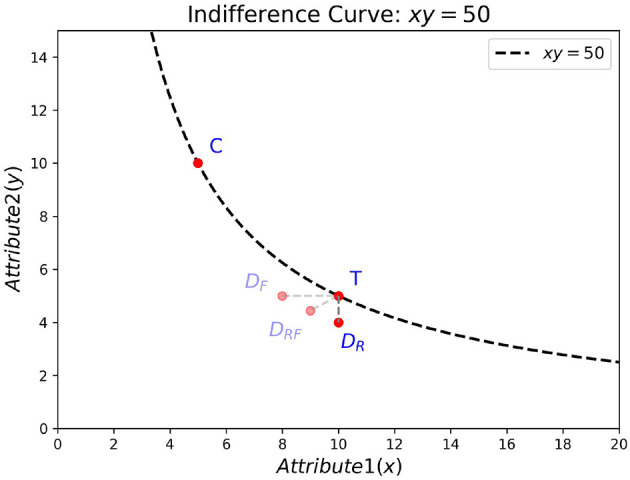
Example of asymmetric dominance in a two-attribute space. The dashed curve shows the indifference curve (*xy* = 50). Items *C* and *T* lie on the curve (area = 50). Three possible decoys are shown: *D*_*F*_ (frequency decoy), *D*_*R*_ (range decoy), and *D*_*RF*_ (range-frequency decoy).

Figure 1 illustrates an example set of items located in a two-dimensional attribute space (*x* and *y*), where the criterion value is given by the product of the attributes (i.e., *x*×*y*). Here, item *C* represents a rectangle with width = 5 units and height = 10 units, whereas item *T* represents a rectangle with width = 10 units and height = 5 units. Assuming that the two rectangles are perceived as equivalent in area (each having 50 square units), a decoy is an item that is inferior to the target in one or both attributes and thereby favors the target. If the decoy is inferior to the target in the target's strongest attribute, it is called a frequency decoy (*D*_*F*_); if it is inferior to the target in the target's weakest attribute, it is called a range decoy (*D*_*R*_). If the decoy is inferior to the target in both attributes, it is called a range-frequency decoy (*D*_*RF*_) (Huber et al., 1982). For simplicity, we consider here *D*_*R*_, which corresponds to a rectangle with an area of 40 square units.

Imagine we ask subjects to rank-order the items along one attribute dimension at a time. Irrespective of whether they do an ordinal comparison of two or three items at a time, the decoy in our example will be asymmetrically dominated according to the attribute-based definition, since it does not surpass the target on either attribute but is at least as good as, or better than, the competitor on one attribute dimension. In contrast, if we present pairs of items at a time and ask subjects to make choices based on the overal criterion values, and they integrate the two attributes into a common currency (here, area), the same decoy will no longer be asymmetrically dominated (the item-based definition). Instead, it will be symmetrically dominated by both the target and the competitor (each with an area of 50 compared to the decoy's 40).

### 1.2 Pairwise comparisons

Sequential sampling models have provided a rich framework for understanding multi-alternative, multi-attribute choice by viewing decision making as a process of repeated comparisons among options and attributes, with attention shifting to compare and evaluate subsets of options throughout the deliberation process (Roe et al., 2001; Usher and McClelland, 2004). Prominent models of choice (Russo and Dosher, 1983; Wollschläger and Diederich, 2012; Kornienko, 2013; Trueblood et al., 2014; Ronayne and Brown, 2017; Noguchi and Stewart, 2018; Evans et al., 2021) assume that this subset is a pair—pairwise comparisons are at the heart of these models. Eye-tracking studies (Noguchi and Stewart, 2014) also suggest that alternatives are compared in pairs, often along a single attribute dimension.

We primarily draw inspiration for our asymmetric dominance thesis from the stochastic ordinal comparison model introduced by (Srivastava and Schrater 2015). Their framework formalizes decision-making as a Bayesian inference problem, where the relative desirability of an option is computed from past choice histories across varying contexts. In this setting, the model infers probabilistic desirabilities by considering the likelihood that an item would be chosen in each observed subset of options, with attentional weights operationalized as the probability of encountering each context. Rather than assigning exogenous utility values, the model learns from observed pairwise choice outcomes and histories to update its beliefs about the desirability of each item, yielding measurable choice probabilities that reflect context-sensitive and history-dependent preference structures.

For a simpler illustration, we present a non-stochastic version of the ordinal pairwise comparison model, formally defined as follows. Consider a set of alternatives {*A*_1_, *A*_2_, …, *A*_*m*_}. Each alternative is evaluated by comparing it pairwise with every other alternative. For any comparison between *A*_*i*_ and *A*_*j*_ (*i*≠*j*), the model assigns to *A*_*i*_ a score *V*_*i*_(*A*_*i*_, *A*_*j*_), where *V*_*i*_(*A*_*i*_, *A*_*j*_) = 1 if *A*_*i*_ wins, *V*_*i*_(*A*_*i*_, *A*_*j*_) = 0 if *A*_*j*_ wins or there is a tie. The total evidence for *A*_*i*_ is then


$$V_{i}=\underset{j\neq i}{\sum }V_{i}\left(A_{i},A_{j}\right),$$

and the selected alternative is


$$A^{*}={\mathrm{argmax}}_{i}V_{i}\;\text{(the alternative with highest}V_{i}\text{)}.$$

To accommodate cases in which some pairs are compared more frequently or are given greater attentional weight, we introduce non-negative weights *n*_*ij*_ for each ordered pair (*A*_*i*_, *A*_*j*_), yielding the generalized evidence function


$$V_{i}=\underset{j\neq i}{\sum }n_{ij}V_{i}\left(A_{i},A_{j}\right).$$

Table 1 presents the simplest case with *n*_*ij*_ = 1 for all *i*≠*j*, in which each pair is compared once. This highlights how cumulative pairwise victories can capture asymmetric dominance and thereby influence the final choice. This formulation is descriptive: it specifies how aggregate outcomes arise through the sequential, weighted aggregation of pairwise wins, without committing to any particular psychological or computational process that generates the individual scores *V*_*i*_(*A*_*i*_, *A*_*j*_). Each computation may involve comparisons along single attribute dimensions or holistic evaluations, depending on the structure and commensurability of the attributes. By leaving the basis of comparison unspecified, the model remains general and applicable across contexts. Moreover, the framework allows direct experimental manipulation of the attentional weights *n*_*ij*_, while keeping the aggregation rule itself agnostic to the underlying comparison process.

**Table 1:** Pairwise “votes” for ordinal comparison scenarios: (A) AE occurs; (B) No AE.

	**Pairwise contest**	**Winner**	**Points to T**	**Points to C**
(A) AE	T vs. D	T	+1	0
	T vs. C	Tie	0	0
	C vs. D	D or Tie	0	0
	Total		1	0
(B) No AE	T vs. D	T	+1	0
	T vs. C	Tie	0	0
	C vs. D	C	0	+1
	Total		1	1

Other influential models in the literature provide complementary perspectives on pairwise comparison and attention. (Noguchi and Stewart 2018) propose the Multialternative Decision by Sampling (MDbS) model, where decision makers randomly sample attribute-option pairs and make ordinal judgments. Process-tracing data indicate that gaze shifts between pairs drive context effects through differences in winning frequencies. MDbS emphasizes attribute-wise ordinal sampling but does not explicitly model asymmetric dominance at the item level.

The Attribute Commensurability and Context Effects (ACE) model, proposed by (Hayes et al. 2024) highlights how the comparability of attribute scales influences attentional allocation and, consequently, context effects. Incommensurable attributes produce salient, discriminable comparisons, resulting in robust context effects, whereas commensurable scales lead to holistic processing and attenuated effects. This work formalizes an important link between attribute presentation, attention, and context effects, though asymmetric dominance is operationalized indirectly via attention modulation.

(Cataldo and Cohen 2019) offer a flexible comparison-process framework that explains attraction, compromise, and similarity effects by how attentional focus shifts between dimension-level and alternative-level comparisons based on presentation format. Their account emphasizes how stimulus format affects cognitive comparison strategy, but does not treat asymmetric dominance as a mandatory structural condition.

Finally, (Trueblood et al. 2022) introduce an attentional dynamics model that captures both attraction and repulsion effects through flexible allocation of attention in multi-attribute choice. Their model suggests that repulsion effects emerge when attention disproportionately focuses on dissimilar alternatives, reversing expected dominance patterns. This highlights the dynamic nature of attention but does not explicitly embed asymmetric dominance as a fixed principle.

Together, these models underscore the importance of pairwise comparisons and attentional weighting in explaining context effects. The (Srivastava and Schrater 2015) model uniquely integrates asymmetric dominance as a core component while allowing attention to operate independently, providing the theoretical foundation for our asymmetric dominance thesis. Other models may be extended to include explicit dominance asymmetry, which remains an avenue for future work.

### 1.3 Overview of experiments

The current investigation systematically explores the role of item-level asymmetric dominance and attentional modulation in driving the attraction effect across four experiments.

Experiment 1 serves as a diagnostic test to establish dominance asymmetry in binary (pairwise) comparisons. We introduce a novel class of star-shaped stimuli designed to reliably produce robust dominance asymmetry, comparing their performance against traditional rectangular stimuli known to produce inconsistent or reversed attraction effects (Spektor et al., 2018).

Building on these pairwise dominance findings, Experiment 2 tests whether the star stimuli elicit a classic attraction effect in a triplet choice context, assuming the same underlying process in both pair and triplet choice context.

Experiments 3 and 4 address a long-standing puzzle in the perceptual literature: why traditional rectangular stimuli produce positive attraction effects when arranged linearly but reversed effects when arranged in triangular configurations. Experiment 3 investigates this effect through a controlled replication, while Experiment 4 systematically manipulates spatial layout to test attentional mechanisms implicated in modulating evidence accumulation.

Collectively, these studies provide a comprehensive empirical test of the boundary condition that asymmetric dominance of the decoy, coupled with attentional sampling processes, governs the presence and magnitude of attraction effects in perceptual choice.

## 2 Experiment 1

### 2.1 Introduction

In the pre-registered within-subjects experiment 1, we used two independent variables—stimulus type (rectangle vs. star) and comparison pair (CD vs. TD). We predicted a significant interaction effect on the dependent variables of accuracy and reaction times. In addition, we collected perceived difficulty ratings of choice (exploratory) when alternatives were compared in pairs.

### 2.2 Method

#### 2.2.1 Participants

Sixty-seven (15 female) university students with normal or corrected-to-normal vision (M: 21.57 years, SD: 2.88, range: 17–31), participated in the study.

#### 2.2.2 Apparatus and stimuli

The experiment was designed using JavaScript and conducted on laboratory computers with screen resolutions of 1920px × 1080px and a screen size of 24 inches (16:9). Stimuli were presented in pairs (target-decoy pairs and competitor-decoy pairs), and consisted of two types of shapes: rectangles and star-like shapes.

In each trial, the stimuli consisted of two horizontally aligned black-colored shapes on a white background. The two stimuli were positioned horizontally around the screen center at 30% and 70% of the total screen width (*x* = 576 px and *x* = 1344 px, respectively), separated by 768 px (≈21.3cm;≈20.1° of visual angle at a 60cm viewing distance). The vertical positions of the stimuli were jittered across trials. The stimulus pair for each trial was derived from the respective set of triplets, which in turn were created in steps from the core stimuli. The term core stimuli refers to the two base stimuli forming the primary comparison set in each trial–the Target (T) and Competitor (C). These rectangular shapes were pre-generated and matched on the relevant decision criterion (equal area) to ensure baseline equivalence before constructing decoy variations. Decoy stimuli (D) were subsequently derived from these core shapes by systematically adjusting one or both dimensions. In each trial, either a target-decoy pair or a competitor-decoy pair was displayed.

##### 2.2.2.1 Rectangle stimulus

To generate the rectangle-based stimuli, we first created a set of 6 random rectangles (the “W” set, wide) by sampling from a bivariate normal distribution (mean height: 170 px, mean width: 250 px; standard deviation: 25 px for each attribute, no correlation), following (Spektor et al. 2018). To create matched but vertically oriented rectangles (the “N” set, narrow), the width and height of the W set were swapped for each pair, preserving area but altering orientation. This resulted in 6 core pairs, each consisting of a wide (W) and a narrow (N) rectangle.

For each of the 6 core pairs, we randomly assigned one of three trial types (range, frequency, range-frequency) (Huber et al., 1982), with each trial type appearing twice across the 6 pairs. Following the triplet-triplet design by (Wedell 1991), for every pair, two sets of triplets were generated: (1) with W as the target (T) and N as competitor (C), and (2) with N as the target and W as competitor. For each triplet, a decoy rectangle (D) was constructed based on trial type manipulation rules (by reducing width and/or height by 10 to 25 px relative to the target rectangle, according to the assigned trial type).

This procedure yielded 12 triplets (6 W-target, 6 N-target), each consisting of a T, C, and D rectangle. For each triplet, two experimental trials were constructed by presenting either the Target-Decoy (TD) pair or the Competitor-Decoy (CD) pair. Thus, the rectangle stimulus set resulted in a total of 24 experimental trials per participant.

Quantitatively, the resultant target-decoy distance—measured as the relative difference in *criterion values* considering both attributes—had a mean of 11.36% (SD = 2.17%) and ranged from 8.43%–14.71%. The distribution was approximately symmetrical without strong skewness.

##### 2.2.2.2 Star stimulus

A parallel procedure was followed for star-shaped stimuli. Each star-like shape was derived from a base rectangle, with four distinct sections removed. These sections consisted of two pairs of inward-facing isosceles triangles, where the bases of the triangles were equal to and touched the four sides of the rectangle, resulting in a star-like shape.

The shape characteristics were determined by two key parameters: the base rectangle's width and the height of the removed triangles. Specifically, for the first out of the two sets of core stimuli, the mean rectangle width (μ_*w*_1__) was set to 180 px, while the mean triangle height (μ_*d*_1__) was set to 40 px. The respective variances were 30 px ($\sigma_{w_{1}}^{2}$) and 40 px ($\sigma_{d_{1}}^{2}$), with no correlation between the two.

Additionally, the height *H*_1_ for the shape was determined by adding a random adjustment to the width. This adjustment was drawn from a normal distribution with a mean of 20 px and a standard deviation of 5 px. The value of *H*_2_, representing the second height, was set equal to *H*_1_.

The participants were instructed that the shapes represented objects drawn with sand, and their task was to identify which of the given shapes would require the least amount of extra colored sand to be extended into a perfect square. Of the two core shapes, one had a wider base rectangle and a larger removed triangle height, while the other had a narrower base rectangle and a proportionally smaller removed triangle height. For simplicity, we refer to the wider shape as “W” (wide) and the narrower shape as “N” (narrow).

To generate the stimuli, the W shapes were created first using the width and height distributions mentioned above. Then, ensuring that both W and N shapes required the same amount of extra area to form a perfect square, the N shapes were derived. As with rectangles, 6 core pairs of W/N stars formed the basis of the stimulus set. Each core pair was randomly assigned one of three trial types (range, frequency, range-frequency), such that all types appeared twice. For each pair, both possible target-competitor arrangements (W as target, N as target) were used. Corresponding decoy stars were created by manipulating the base rectangle's width (reduced by 8-15 px) and the height of the removed triangles (increased by 4-7 px) relative to the target, according to the assigned trial type. Thus, there were 12 triplets (6 W-target, 6 N-target), each producing two experimental trials (TD and CD). This resulted in 24 experimental star trials per participant.

The target-decoy distance–measured as the relative difference in *criterion values* considering both attributes—had a mean of 13.97% (SD = 2.14%) and ranged from 10.53% to 16.91%. The distribution was approximately symmetrical without strong skewness.

Additionally, 12 catch trials were included throughout the experiment. In these trials, participants always received the instruction: “Choose the shape that requires the least amount of extra colored sand to make it a perfect square.”—the same instruction as for star trials. Catch trials presented vertically oriented rectangles pairs designed so that one option clearly required less extra sand to complete into a square than the other, varying in only one attribute (width for rectangles).

To summarize, each participant completed 24 rectangle-based trials, 24 star-based trials, and 12 catch trials, for a total of 60 trials. For rectangle stimuli, the criterion value was defined as the area (width × height). For star stimuli, the criterion value was the area of additional “sand” required to complete the shape into a perfect square, calculated using the geometric parameters of the base rectangle and the removed triangles. The OSF repository contains the exact stimulus set. In the Supplementary Section 1, we present further details on the star stimuli creation with the full formula for criterion value matching.

Although the rectangle and star stimuli involved different surface attributes (rectangles: width and height; stars: base width and height of the removed triangular section) and corresponded to different judgment tasks (estimating overall area vs. estimating sand required to complete a square), in both cases, stimulus variation was systematically governed by two orthogonal attributes. Importantly, decoy manipulations were constructed within the same underlying attribute framework, ensuring consistency in the experimental implementation of the attraction effect across the two stimulus types.

#### 2.2.3 Design

The experimental conditions were defined by two independent variables: (1) Stimulus Type (rectangle vs. star), and (2) Comparison Pair (Target-Decoy [TD] vs. Competitor-Decoy [CD]). In a within-subjects design, each participant experienced all four combinations of these variables: (1) Rectangle, CD, (2) Rectangle, TD, (3) Star, CD, and (4) Star, TD.

The trials were presented using block randomization to ensure balanced exposure to all conditions. The experiment consisted of 12 blocks, each containing one trial per condition. Each condition was presented 12 times, resulting in 48 experimental trials. The Fisher-Yates algorithm was applied to randomize the order of conditions within each block, ensuring that the sequence of trials was unpredictable while maintaining balance. Additionally, 12 catch trials were included as exclusion criteria and were randomly interspersed throughout the experiment. The catch trials were distributed across the trial sequence using the same randomization method, ensuring a unique and unbiased presentation for all participants.

Each trial comprised a choice response phase followed by a rating task, with no time constraints imposed. The number of trials per participant was selected to balance robust data collection with participant comfort. Our design maximized data quality and engagement while minimizing fatigue, in light of the absence of imposed time constraints and the inclusion of additional rating tasks in each trial. Moreover, recent simulation-based evidence (Miller, 2024) demonstrated that increasing participant sample size typically contributes more to statistical power than a dramatic increase in trial numbers, especially when longer experimental durations risk inducing fatigue or noncompliance. Reduced trial load thereby supports better data reliability and sustained task engagement in within-subject perceptual and decision-making paradigms. Additionally, stimulus randomization and block balancing procedures ensured full counterbalancing of all relevant conditions, supporting robust statistical inference despite a modest number of trials per participant.

#### 2.2.4 Procedure

At the beginning of each trial, a fixation cross was presented for 500 ms. This was immediately followed by the stimulus display, which remained on the screen until the participant responded via key press–there was no time limit imposed for the choice response. Upon making a selection, a rating task appeared, which similarly remained visible until a key press response was recorded. There was no time restriction for ratings. After each trial, the next fixation cross was presented for 500 ms before the onset of the following trial.

For rectangle trials, participants were instructed to select the rectangle with the largest area. For trials with star-like shapes, they were instructed to choose the shape requiring the least amount of additional colored sand to extend it into a perfect square. Participants selected the alternative in each trial using the left or right arrow keys. Following their decision, using number keys 1–7, they rated the difficulty of their choice on a 7-point Likert scale, where one represented “extremely easy” and seven represented “extremely difficult”.

Before the main experiment, each participant completed a feedback-based practice session which involved only star stimuli. These practice trials ensured comprehension of the more complex stimulus evaluation instructions. No separate practice was required for the rectangular stimuli, as participants readily understood and performed this task during pilot testing. In the practice session, the participants were presented with 10 pairs of star shapes in random order. In five trials, the W stimulus was the expected answer, and in five trials, the N stimulus was the expected answer. Participants could click on each black shape to transform it into a perfect square, with the extra-filled portion highlighted in red. When a shape was clicked, a numerical value representing the extra sand required (e.g., 21,458 units) appeared below the shape in an arbitrary unit of measurement. The practice session was to ensure that participants understood the task instructions, especially for the star task.

### 2.3 Results

Out of a total of 67 participants, we excluded data from 6 participants because their performance was lower than 0.8 in the catch trials, where in each trial, there was clearly one best option out of the two. Additionally, we excluded a total of 119 individual trials (4.06%) that were either too fast (< 100 ms) or too slow (>20,000 ms).

**Figure 2:**
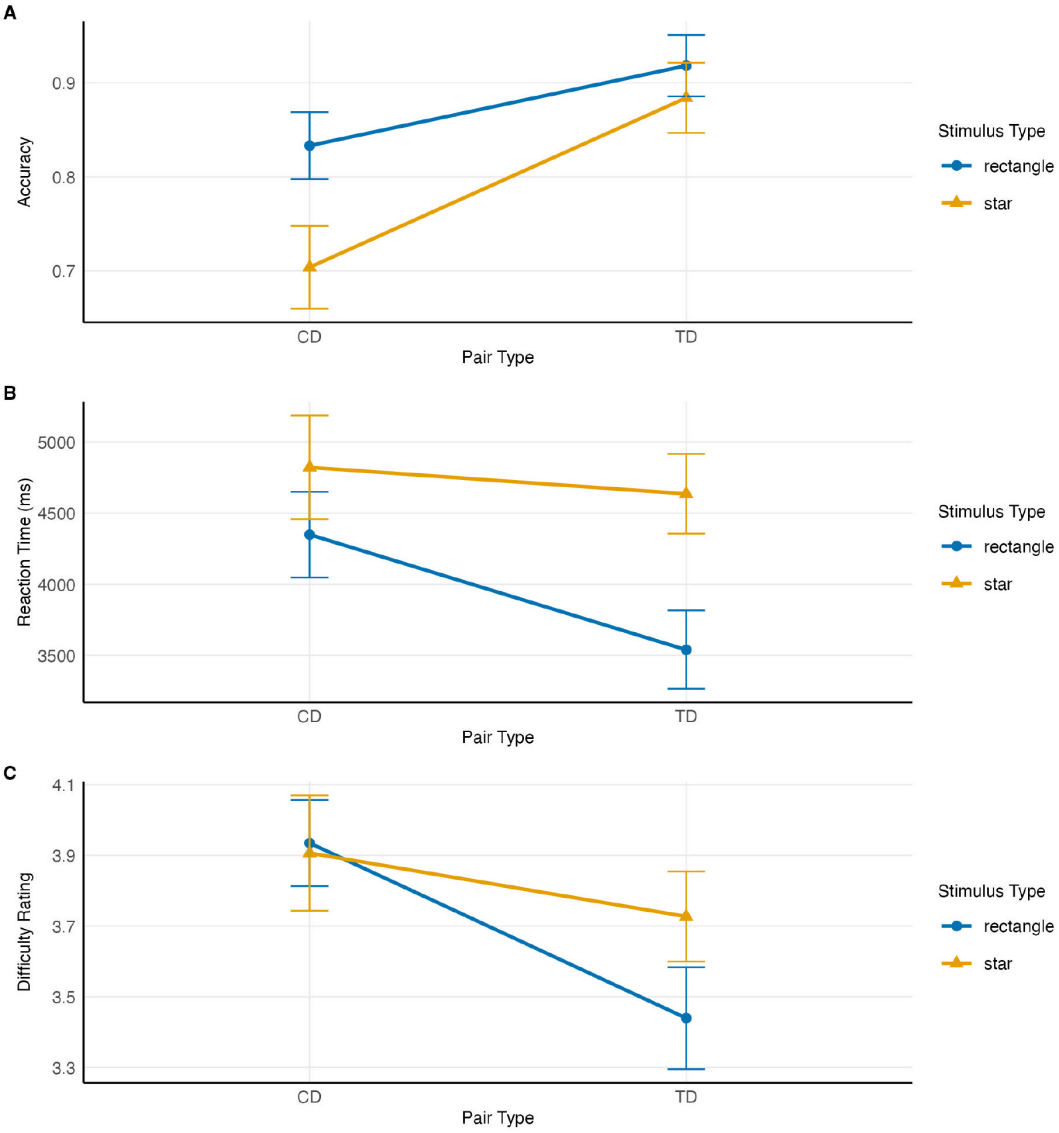
Interaction effects of stimulus type and pair on **(A)** accuracy, **(B)** reaction time, and **(C)** difficulty. Error bars represent 95% within-subject confidence intervals.

We performed repeated measures ANOVAs on accuracy, reaction time, and perceived difficulty ratings. The interaction effects of *Stimulus Type* (Star vs. Rectangle) × *Comparison Pair* (CD vs. TD) on all three were significant. ANOVA results are in Table 2. Interaction plots are shown in Figure 2. The main effects of the pair were significant for difficulty rating, accuracy, and RT. On average, the CD pair was rated as more difficult than the TD pair, and performance (both accuracy and RT) was better for the TD pair than the CD pair. Similarly, the main effects of stimulus type were also significant for accuracy and RT. Performance for rectangular stimuli (both RT and accuracy) was better compared to star stimuli. However, considering both the stimulus type and the comparison pair interaction, the pattern of results diverged between the stated difficulty and the revealed difficulty (as measured by accuracy), as well as RT.

**Table 2:** Repeated measures ANOVA results for accuracy, reaction time, and difficulty rating.

**Factor**	***F*(1, 60)**	***p***	** $\eta_{p}^{2}$ **
**Accuracy**			
Stimulus type	11.06	0.002	0.16
Pair	75.82	< 0.001	0.56
Stimulus type × Pair	9.81	0.003	0.14
**Reaction time**			
Stimulus type	27.08	< 0.001	0.31
Pair	9.62	0.003	0.14
Stimulus type × Pair	4.47	0.039	0.07
**Difficulty rating**			
Stimulus type	2.50	0.119	0.04
Pair	22.82	< 0.001	0.28
Stimulus type × Pair	8.77	0.004	0.13

Each row reports the *F* value, *p* value, and partial eta squared ($\eta_{p}^{2}$) for the corresponding main effect or interaction.

In the *post hoc* analysis, the accuracy difference between the two pairs was significant for the star stimuli (*t*(60) = 7.079, *p* < 0.001, Cohen's *d* = 1.016, mean difference = 0.180, SD = 0.199). In contrast, the TD CD accuracy difference for rectangle stimuli, while still significant, was notably smaller (*t*(60) = 5.092, *p* < 0.001, Cohen's *d* = 0.576, mean difference = 0.085, SD = 0.130). Moreover, a pairwise *t*-test between the delta accuracies (TD–CD) for the two stimulus types (star vs. rectangle) revealed a significant difference, *t*(60) = 3.132, *p* = 0.003, Cohen's *d* = 0.567.

To ensure that the observed differences between stimulus types were not driven by variability in the distribution of %Target–Decoy distances (TD_dist%), we conducted a supplementary sensitivity analysis that included only trials where the TD_dist% ranges overlapped for both rectangular and star stimuli. The results of this analysis closely mirrored those derived from the full dataset, confirming that the reported effects reflect genuine stimulus-type differences rather than artifacts of distributional variation (see Supplementary Section 2, for details). Additionally, robustness checks that varied participant exclusion thresholds and trial-level RT cutoffs (Supplementary Table S5) revealed that the observed pattern of results for accuracy remained stable across all tested criteria.

### 2.4 Discussion

Using accuracy as a proxy for decoy's dominance, the results support our hypothesis regarding the asymmetry of dominance, at least in the pairwise comparisons. The strong main effect of pair in the accuracy results reveals a differential accessibility of dominance relations between TD and CD pairs. This likely arises because the Target and Decoy (T and D) are close in attribute space, affording easier ordinal comparisons–often directly along a single attribute dimension. In contrast, the Competitor and Decoy (C and D) are placed farther apart in the attribute space, such that each alternative tends to be superior on different attributes: one is stronger on one attribute, while the other excels on another. This configuration reduces the ease of establishing clear ordinal dominance in CD pairs, requiring attention to trade-offs or joint evaluation across attributes.

The interaction effect in the accuracy results suggests that the decoy's dominance asymmetry was strong, though not ideal, for star stimuli but less pronounced for rectangle stimuli. To rule out the possibility that these results were influenced by a baseline preference bias for either wide (W) or narrow (N) core options, we fit a linear mixed-effects model including target type and its interaction with pair type. The interaction was not significant (*p* = 0.54), confirming that the dominance asymmetry was robust across both W and N targets (see the Supplementary Section 3 for full details).

Surprisingly, for self-reported difficulty and RT, there was a difference between CD and TD only with rectangular stimuli. It is not clear why the less accurate star stimuli do not show a significant difference in RT and perceived difficulty between the CD and TD pairs. One possible explanation is that the star stimuli, by virtue of their more complex and novel geometry, were generally perceived to be at a higher level of task difficulty that reduced participants' sensitivity to subtle pairwise differences. This increased overall challenge may have led to greater variance both within and across participants, thereby masking any RT or perceived difficulty effects that would otherwise mirror the strong asymmetry observed in accuracy. Alternatively, the star task may have encouraged a more deliberative or strategy-driven mode of processing, such that participants did not rely on “fast” ease-of-comparison cues to the same extent as with familiar rectangles. This difference in cognitive approach could dampen RT and subjective difficulty differences, even as underlying choice asymmetries remain robust. Future work could probe these possibilities by using eye-tracking or confidence ratings to directly compare processing dynamics across stimulus types.

Note that while the star stimuli produced robust asymmetric dominance, with participants reliably preferring the target over the decoy in TD pairs but not showing similar choice in CD pairs, rectangles also showed significant, though weaker, asymmetry. Thus, although stars provide a stronger diagnostic of dominance asymmetry, even rectangles cannot be considered entirely free of item-level asymmetry.

Having established that star stimuli yield a particularly clear dominance structure, we next asked whether this advantage would carry over to triplet choice sets. Experiment 2 therefore tested whether the newly developed star stimuli elicit a robust attraction effect in a triangular triplet arrangement, addressing prior reports of null or even reversed effects with perceptual stimuli.

## 3 Experiment 2

### 3.1 Introduction

Building on the results of Experiment 1, which showed that star stimuli produce strong asymmetric dominance in pairwise comparisons, we next asked whether this property would carry over to triplet choice sets. The assumption was that the underlying cognitive process producing asymmetric dominance in pairwise comparisons would also operate in the case of triplets and would lead to a positive attraction effect. Experiment 2, therefore, tested whether the novel star-shaped stimuli generate such an effect in the standard triangular configuration used in decoy paradigms.

### 3.2 Method

#### 3.2.1 Participants

Fifty-four students (12 female) with normal or corrected-to-normal vision (M: 20.17 years, SD: 3.08, range: 17–32), participated in the study.

#### 3.2.2 Apparatus and stimuli

The experiment was designed using JavaScript and conducted on similar laboratory computers. In each trial, the stimuli consisted of three different black-colored star shapes on a white background. The three stimuli (target, competitor, and decoy) were simultaneously displayed at the vertices of a virtual, upright equilateral triangle centered on the display, with their vertical positions jittered across trials. The base of the triangle was defined horizontally by points located at 30% and 70% of the total screen width, corresponding to pixel coordinates *x* = 576 and *x* = 1, 344 on a 1920-pixel-wide monitor—yielding a base length of 768 pixels. The height of the triangle was calculated as $h=\left(\sqrt{3}/2\right)*s$, where *s* is the base length, resulting in a height of approximately 665 pixels. The top vertex was located at the horizontal midpoint of the display (*x* = 960), vertically positioned 665 pixels above the center of the base line, i.e., at *y* = −125 relative to screen center (*y* = 540). The screen coordinates are defined relative to the screen center (0, 0), with positive y values extending downward.

At a fixed viewing distance of 60 cm on a 24-inch monitor (16:9), the triangle base corresponded to 212.5 mm (20.1° visual angle), and its height to 184.1 mm (17.4° visual angle). Thus, the left vertex was at (576, 540), the right at (1344, 540), and the top vertex at (960, −125), ensuring consistent and well-controlled spatial separation for all stimulus triplets across trials.

The star-like shapes were constructed following the method used in Experiment 1. Each star-like shape had the width of the base rectangle and the height of the removed triangles as its two attributes.

#### 3.2.3 Design

Experiment 2 employed a forced-choice design in which participants made a selection among three simultaneously presented star-like shapes, constructed to differ systematically along two attribute dimensions: the width of the base rectangle and the height of the removed triangles.

In addition to the transition from pair to triplet comparisons, Experiment 2 incorporated a bias correction that distinguished it from Experiment 1. A pilot study revealed a systematic preference among participants for the wider (W) shape. To counteract this bias in the main experiment, the computed width of the narrower (N) shape—denoted as *w*_2_—was increased by 10 pixels (see Supplementary Section 4). This adjustment aimed to equalize the perceptual appeal of the stimuli and ensure a more balanced choice distribution.

A total of 180 trials were administered to each participant, which took approximately 40 minutes to complete. Of these, 144 were main trials, equally divided between 72 trials with the wider star shape (W: wide) as the target and 72 trials with the narrower star shape (N: narrow) as the target. Additionally, 36 catch trials were included to assess participant-level exclusion criteria. Similar to Experiment 1, each participant completed a feedback-based practice session with 10 trials before the main experiment.

This experimental design was chosen to maximize control over stimulus presentation, enable robust counterbalancing of all conditions, and facilitate reliable statistical inference, while also accounting for participant fatigue and engagement.

#### 3.2.4 Procedure

At the start of each trial, a fixation cross appeared at the center of the screen for 500 ms. Immediately thereafter, the three star-like stimuli were displayed in a triangular arrangement on a white background. The stimulus display remained visible until participants made a choice response using the arrow keys; there was no imposed time limit for this response. Upon selection, participants proceeded to a rating task, which also remained visible until a response was made via key press, with no time restriction. After each trial, a new fixation cross appeared for 500 ms before the onset of the next trial.

During the practice session, participants were provided feedback following each selection to help familiarize them with the task. The main experiment did not include feedback. This study was not preregistered.

### 3.3 Results

Data were collected from 54 participants. First, 11 participants were excluded due to a technical error resulting in missing response time (RT) data for the majority of their trials; for these participants, only the first 10 trials contained RT data, while the remaining 170 trials were missing. This technical exclusion ensured data quality and left 43 participants for further analysis. Subsequently, one participant who failed to meet the predefined catch trial accuracy threshold of 0.8 was excluded, resulting in a final sample of 42 participants.

Within the retained sample, all catch trials were removed from further analysis. Individual trials were further excluded if their RT was less than 100 ms or exceeded 20,000 ms, excluding 4.07% of main trials and yielding 7,252 valid trials for subsequent analysis.

We quantified context effects using the equal-weights version of the Relative Choice Share of the Target (RST), as defined by (Katsimpokis et al. 2022), for a triplet-triplet design (Wedell, 1991). This measure captures how often the target is chosen over the competitor, where an RST of 0.5 denotes no context effect. Formally,


$$RST_{\text{EW}}=\frac{1}{2}\left(\frac{T_{X}}{T_{X}+C_{X}}+\frac{T_{Y}}{T_{Y}+C_{Y}}\right)$$

where *T*_*X*_ and *C*_*X*_ represent the target and competitor selections when the decoy favors option X, and *T*_*Y*_ and *C*_*Y*_ represent these counts when the decoy favors option Y. In this context, *X* and *Y* indicate the two core stimuli. Values above 0.5 indicate a bias toward the target. It should be noted that this metric that is widey used for the attraction effect—change in choice probabilities with decoy introduction—is itself an empirical measure of IIA violation, generally called the context effect.

A two-tailed one-sample *t*-test against 0.5 revealed that the mean RST was significantly above chance (*M* = 0.537, *SD* = 0.050); *t*(41) = 4.815, *p* < 0.001, Cohen's *d* = 0.723. Complementary Bayesian *t*-tests using the Jeffreys–Zellner–Siow prior (scale *r* = 0.743) provided strong evidence supporting the alternative hypothesis (*BF*_10_ = 1035.9), indicating that the observed effect is approximately 1036 times more likely under the presence of a positive context effect than under the null hypothesis.

**Figure 3:**
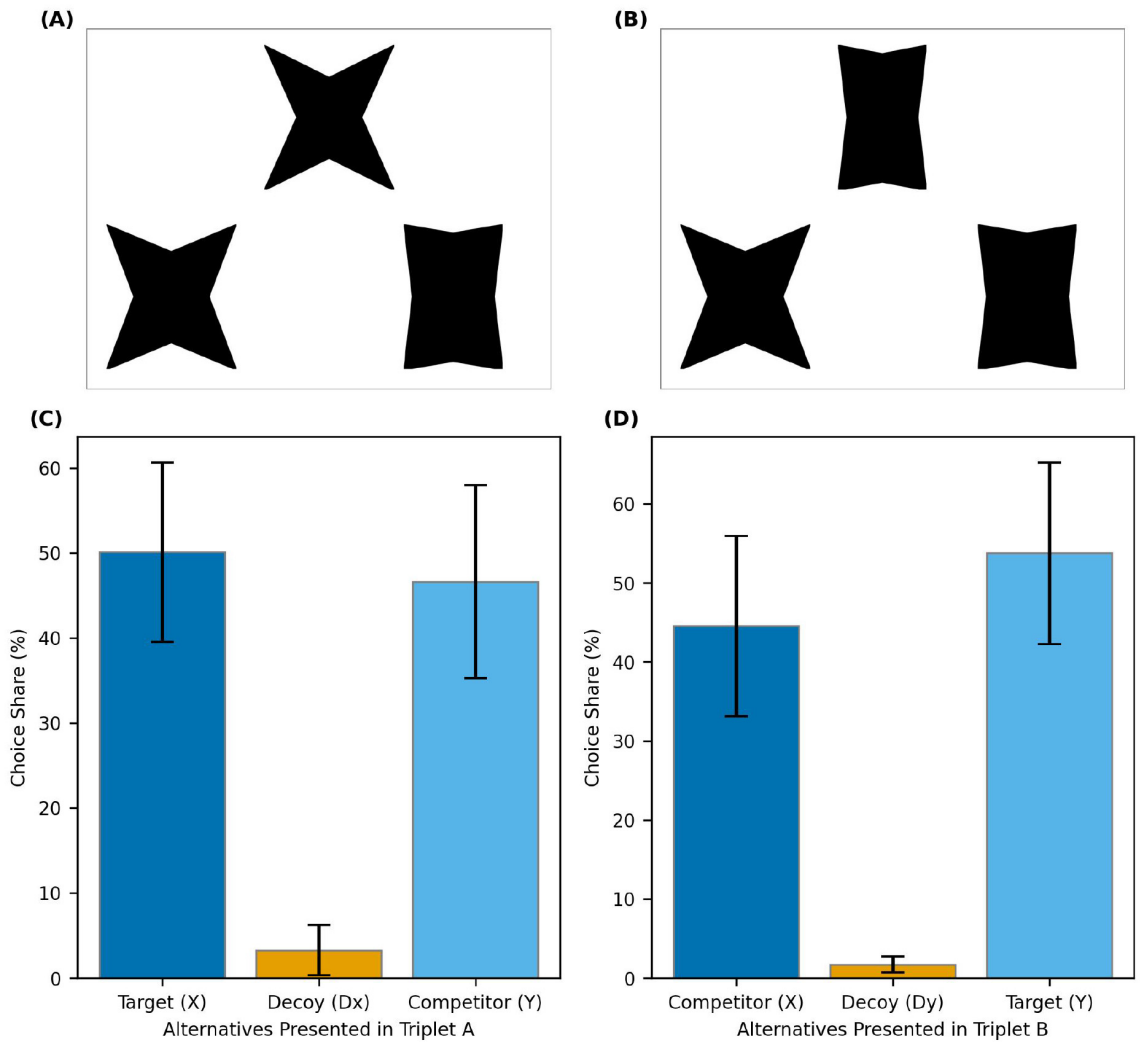
Example trials and choice shares in *Experiment 2*. **(A)** A trial where the wider stimulus serves as the target. **(B)** A trial where the narrower stimulus is the target. **(C)** Choice shares when X is the target and Dx (a decoy inferior to X) is presented. **(D)** Choice shares when Y is the target and Dy (a decoy inferior to Y) is presented. Error bars represent 95% within-subject confidence intervals.

**Figure 4:**
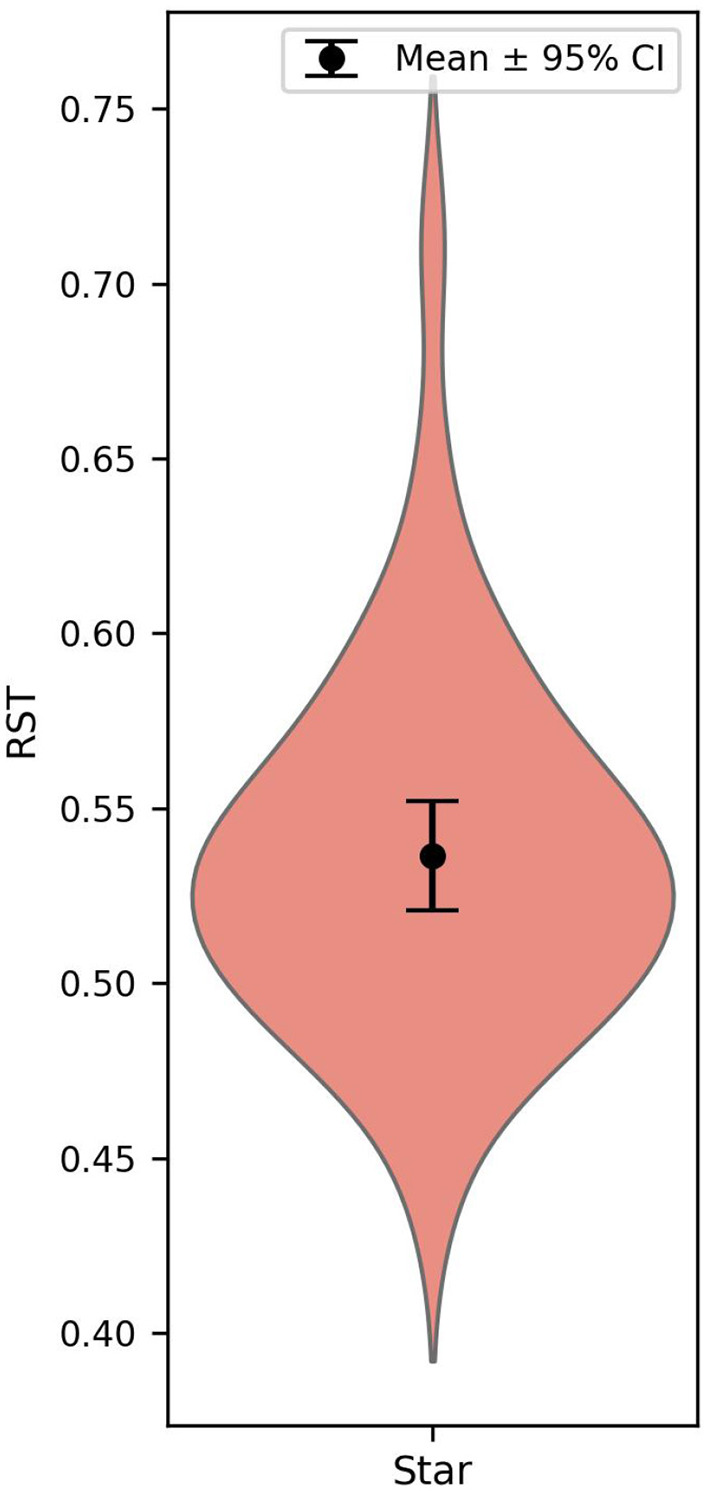
Relative share of the target in Experiment 3. The dot represents the mean, and the error bars indicate 95% confidence intervals based on between-subject variability.

To verify the robustness of these findings, we conducted extensive sensitivity analyses varying participant catch trial accuracy thresholds and trial-level RT exclusion criteria. The results of these analyses are reported in Supplementary Table S6, which shows that the positive context effect remains statistically significant and stable across all tested exclusion permutations. Figure 3 shows two example trials and the overall distribution of the choice share in the two contexts. Figure 4 depicts a corresponding violin plot for the overall RST values. Further descriptive statistics on choice frequencies and response time are reported in the Supplementary Section 7.

### 3.4 Discussion

To our knowledge, this study is the first to demonstrate the positive attraction effect for perceptual stimuli arranged in a triangle. Experiment 2 demonstrated that the newly introduced star stimuli elicit a reliable attraction effect in triplet choice sets. This finding provides direct support for the hypothesis that asymmetric dominance in pairwise comparisons is a necessary precondition for the AE: the same stimuli that produced strong asymmetry in dyadic judgments (Experiment 1) also yielded robust attraction in the triplet format.

Together, Experiments 1 and 2 strengthen the case that perceptual attraction effects depend critically on the presence of clear item-level dominance relations. Having established this principle with novel star stimuli, we next turned to a long-standing puzzle in the literature: why rectangle stimuli, which show reliable AEs in linear arrangements, have sometimes produced reversed effects in triangular configurations. One possibility is that this puzzling outcome reflects the weaker asymmetry in decoy's dominance we observed for rectangles in pairwise tasks. Experiment 3, therefore, tested whether the apparent reversal would persist under replication, or whether it would instead resolve into a reduced–but still positive–attraction effect.

## 4 Experiment 3

### 4.1 Introduction

Experiment 3 addressed the puzzling finding that rectangle stimuli sometimes yield a reversed (negative) attraction effect in triplet choice sets (Spektor et al., 2018). Given that Experiment 1 revealed only weak asymmetric dominance for rectangles in pairwise comparisons, we predicted that in triplet set, the same stimuli should not generate a true repulsion effect but rather a reduced or null attraction effect. To test this, we ran a conceptual replication of the triangular triplet design of (Spektor et al. 2018).

In parallel, we revisited the study by (Spektor et al. 2022), which had reported a repulsion effect with bar stimuli. Their original analysis relied on RST scores collapsed across multiple within-subject manipulations, raising the possibility that the observed effect reflected the interaction of these factors rather than the decoy's role itself. To address this, we reanalyzed their data at the trial level, isolating the decoy manipulation.

The motivation for including both the direct replication and the reanalysis was two-fold. First, both (Spektor et al. 2018) and (Spektor et al. 2022) reported negative attraction effects using perceptual stimuli. We aimed to examine whether multi-attribute perceptual stimuli would produce results consistent with our theoretical framework—that is, whether weak or absent asymmetric dominance would lead to null rather than negative attraction effects. Second, the dataset from (Spektor et al. 2022) is both publicly available and atypical in its multi-factorial design, allowing us to test—within a model comparison framework—whether the negative effect reported originally persists when all design factors are explicitly accounted for.

Our interpretation regarding the absence of strong asymmetric dominance of the decoys in (Spektor et al. 2022) is theoretically motivated, based on their described stimulus construction, but was not directly tested within their dataset. Based on this reasoning, we anticipated that both the replication (Experiment 3) and the subsequent reanalysis would yield null rather than negative effects. Under our framework, when dominance asymmetry is not robustly established, we expected an absence of a context effect (i.e., a true null) rather than a reversal.

### 4.2 Method

#### 4.2.1 Participants

Seventy-six volunteers (19 female) with normal or corrected-to-normal vision (M: 20.93 years, SD: 2.56, range: 17–28), participated in the experiment.

#### 4.2.2 Apparatus and stimuli

This experiment was designed using JavaScript and conducted on the same laboratory computers as in previous experiments. Rectangle stimuli were created following the procedures described in Experiment 1, and the decoy construction method (including range, frequency, and range-frequency decoys) was consistent with Experiment 1. The spatial arrangement and display calibration for stimulus presentation were identical to Experiment 2; that is, on each trial, three stimuli were simultaneously displayed at the vertices of an upright, virtual equilateral triangle centered on the screen (see Section 3.2.2, Apparatus and Stimuli, Experiment 2, for full geometric and display details).

#### 4.2.3 Design

Experiment 3 employed a forced-choice design in which participants made a selection among three simultaneously presented rectangles, each differing in area. The arrangement of rectangles and decoy generation methods (range, frequency, and range-frequency decoys) were matched to those used in Experiment 1 to enable direct comparison across experiments.

A total of 162 trials were presented to each participant. These trials included 144 main trials and 18 catch trials, ensuring all conditions were fully counterbalanced.

The trial count was determined to balance robust data quality with participant comfort and engagement. Randomization of trial presentation ensured that each participant experienced every condition, supporting reliable statistical inference.

#### 4.2.4 Procedure

At the start of each trial, a fixation cross appeared at the center of the screen for 500 ms. Immediately thereafter, three rectangle stimuli were displayed in a triangular formation on a white background. Participants were instructed to select the rectangle with the largest area. The stimulus display remained on the screen until the participant made a choice response by pressing the corresponding key; there was no imposed time limit for this response. After selection, the next trial began following a 500 ms fixation interval.

Practice trials were presented at the beginning of the session, during which participants received feedback after each response to familiarize them with the task requirements. No feedback was provided during the main experiment. This study was not preregistered.

### 4.3 Results

Of the 76 participants tested, two were excluded due to a technical error that prevented complete reaction time (RT) logging. An additional six participants were excluded for performing below the predetermined accuracy threshold (i.e., less than 80%) on catch trials.

At the trial level, responses with implausible RTs were excluded. Specifically, trials with RTs below 100 ms or exceeding 20000 ms were removed. This procedure led to the exclusion of 126 trials, representing approximately 1.14% of the total data, and resulted in 10892 valid trials available for analysis.

**Figure 5:**
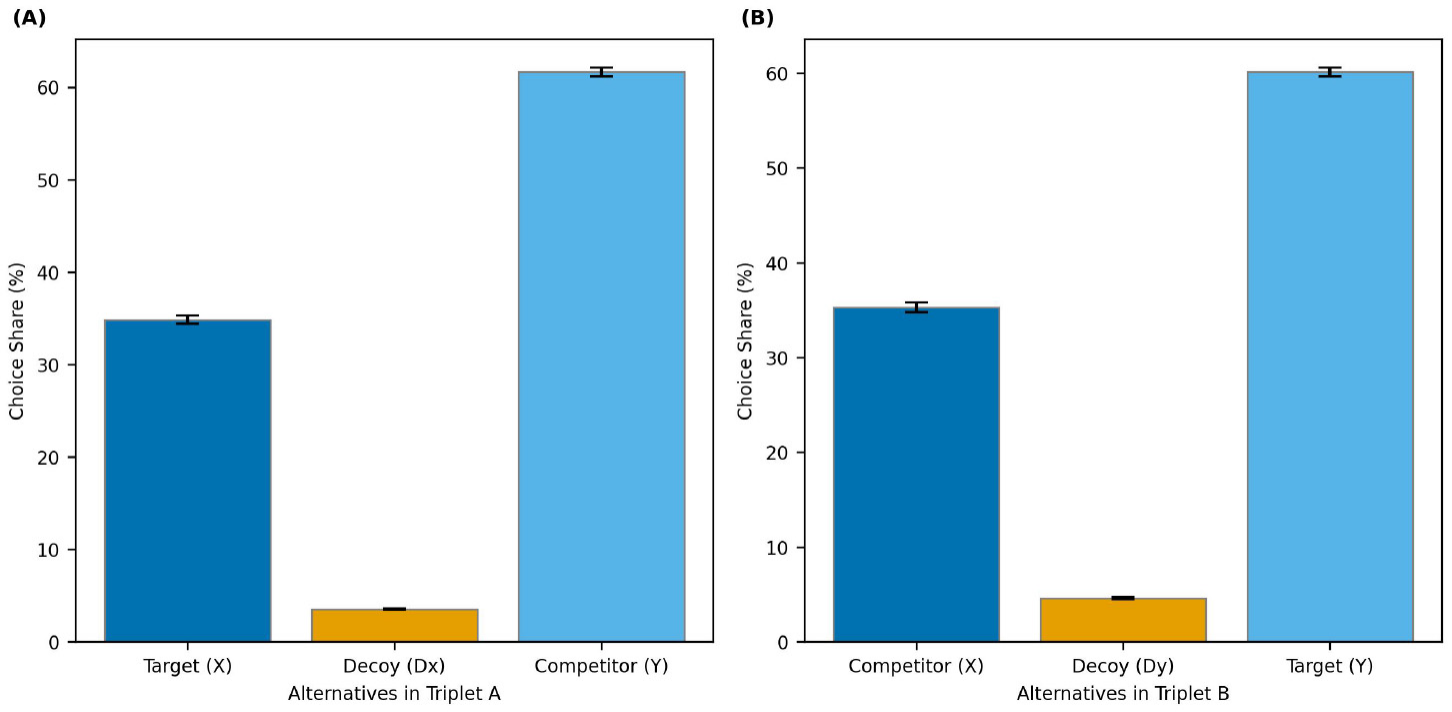
Choice shares in *Experiment 3* for the two triplet contexts. **(A)** Choice shares when X is the target and Dx (a decoy inferior to X) is presented in Triplet A. **(B)** Choice shares when Y is the target and Dy (a decoy inferior to Y) is presented in Triplet B. Error bars represent 95% within-subject confidence intervals calculated using the Cousineau-Morey method.

**Figure 6:**
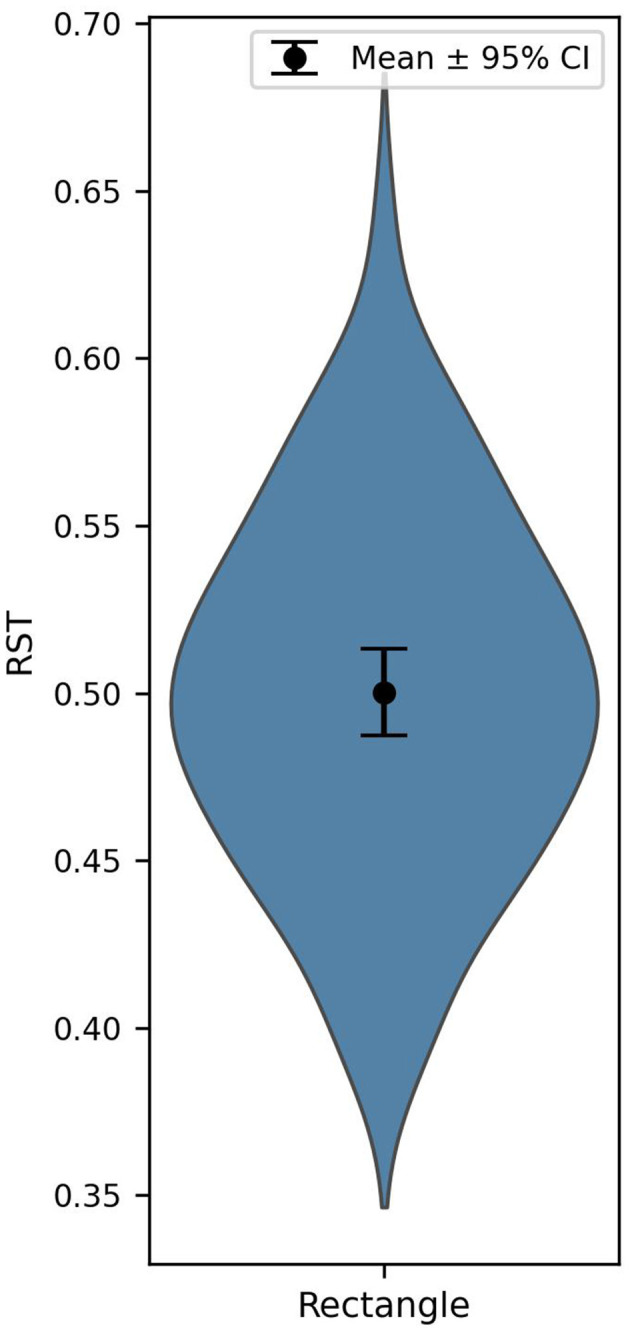
Relative share of the target in *Experiment 3*. The dot represents the mean, and the error bars indicate 95% confidence intervals based on between-subject variability.

We performed a two-tailed one-sample *t*-test on the overall RST. We were unable to replicate the negative AE reported in previous studies; rather, we found that overall RST (*M* = 0.500, *SD* = 0.053) was not significantly different from the null (0.5); *t*_(67)_ = 0.029, *p* = 0.98, Cohen's *d* = 0.004. To further quantify the evidence, we conducted a Bayesian one-sample *t*-test using the default Jeffreys-Zellner-Siow (JZS) prior with a scale parameter *r* = 0.707. The Bayes factor (BF_10_ = 0.13) indicated that the data were about seven times more consistent with the null hypothesis than with the alternative. Figure 5 shows the overall distribution of the choice share in the two contexts, and Figure 6 depicts a corresponding violin plot for the overall RST values. Robustness checks paralleling those conducted in Experiment 2—varying both participant-level and trial-level exclusion thresholds—consistently supported the null hypothesis across all tested parameters (Supplementary Table S7), confirming that the lack of a context effect in Experiment 3 reflects a genuine absence rather than an artifact of data filtering. Further descriptive statistics on choice frequencies and response times are also reported in the Supplementary Section 7.

#### 4.3.1 Re-analysis of an earlier study by Spektor and colleagues

Because bar stimuli have previously yielded reversed attraction effects, we conducted a re-analysis of the original data from (Spektor et al. 2022). That study manipulated several design factors simultaneously, making it difficult to isolate the role of any single manipulation. We therefore compared a set of competing models, including a null model, a baseline additive model, and multiple interaction models. The best-fitting Generalized Linear Mixed-Effects Model (GLMM) yielded a non-significant estimate for the target variable (see Supplementary Section 4 for details). This pattern is most consistent with a null attraction effect.

Experiment 3 tested whether rectangles, which had previously been reported to elicit a negative attraction effect in triangular arrangements (Spektor et al., 2018), would reproduce this pattern. Instead, we observed a null effect: neither a reliable positive nor a negative attraction effect emerged. This finding suggests that the relationship between stimulus geometry and the attraction effect is not straightforward. Rectangles produced weak but significant dominance asymmetries in pairwise comparisons (Experiment 1) and have reliably supported the attraction effect in linear arrangements in prior work (Trueblood et al., 2013), yet in triangular displays they failed to produce the effect.

The re-analysis of (Spektor et al. 2022) provided converging evidence. Although their original report suggested a repulsion effect, this was based on analyses collapsing across multiple within-subject manipulations. By isolating the decoy's contribution with a trial-level GLMM approach, we found no reliable attraction or repulsion effect. While we did not directly test whether the bar stimuli used in their study achieved item-wise dominance asymmetry, our interpretation of these results is theoretically grounded in the assumption–based on their stimulus construction–that such dominance asymmetry was likely weak or absent. Together, the replication and the re-analysis support the conclusion that when dominance asymmetry is not robustly established, the expected outcome is a true null effect, rather than a reversal of the attraction effect.

### 4.4 Discussion

Taken together, the replication and reanalysis reinforce the conclusion that AE depends not only on the presence of asymmetric dominance but also on how spatial arrangements channel attention to target-decoy vs. competitor-decoy relations. A linear layout may enhance the accessibility of these comparisons, whereas triangular arrangements may dilute or redistribute attentional salience across pairs.

Overall, Experiments 1–3 suggest that AE is not guaranteed by perceptual decoys alone: it requires the structural condition of item-based dominance asymmetry. This raises a critical next question: does spatial alignment of options interact with the underlying cognitive processes to produce dominance asymmetry? Experiment 4 was designed to test this possibility.

## 5 Experiment 4

### 5.1 Introduction

Across Experiments 1–2, our findings converged on the idea that the attraction effect (AE) critically depends on the decoy's dominance asymmetry. When decoys created strong asymmetry in pairwise comparisons, as with our novel star stimuli, we observed both clear pairwise asymmetries (Exp. 1) and robust AE in triplet choices (Exp. 2). Moreover, in Experiment 3, the null attraction effects–both in our reanalysis of bar stimuli and in the failed replication of rectangles in triangular displays (Spektor et al., 2018, 2022)–shift the interpretation away from accounts based on a repulsion effect and instead toward a boundary condition defined by the presence or absence of asymmetric dominance. Rectangles presented a more intriguing case: they produced significant asymmetry in pairwise comparisons (Exp. 1) and reliably elicited AE in linear triplet arrangements in prior work, yet failed to do so in triangular arrangements (Exp. 3). This discrepancy raises a key question: why should display layout matter at all?

It is important to note that Experiment 1 involved rectangle pairs presented in linear horizontal alignment. It is also noteworthy that in a triangular arrangement with equal representation of all six possible (T, C, D) configurations, each participant encounters one-third of trials where both TD and CD pairs are oblique, one-third where the TD pair is horizontal and the CD pair is oblique, and one-third where the CD pair is horizontal and the TD pair is oblique. This ensures that in triangular display, the attentional advantages are distributed across conditions, potentially diluting the overall dominance asymmetry. By contrast, linear triplets always contain horizontally aligned pairs, potentially amplifying dominance asymmetry in favor of the target.

One possibility, then, is that linear arrangements introduce systematic attentional biases. For instance, the central item always appears at fixation, and transitions between horizontally adjacent items may be easier than between outer items (Spektor et al., 2022). Moreover, matched orientations in a linear alignment may make target–decoy (TD) pairs consistently salient, regardless of their specific positions. This assumption is reasonable. Indeed, the Multi-attribute Linear Ballistic Accumulator (MLBA) model (Trueblood et al., 2014; Turner et al., 2018; Evans et al., 2019) makes a similar assumption to explain the same positive effect in the linear alignment of rectangle stimuli. According to the model, the relative salience of pairwise comparisons drives the AE: TD comparisons are weighted more strongly than competitor–decoy (CD) comparisons because of attribute similarity, which favors the target. More general frameworks (Trueblood et al., 2022) extend this idea by allowing attentional weights to be modulated by factors such as attribute bias or presentation format. Although these models can retrospectively attribute differences in AE to spatial modulation of attentional weights, existing explanations remain inferential, hinging on untested assumptions about attention allocation.

Psychophysical research strengthens this prediction. Across diverse paradigms, horizontally aligned stimuli enjoy processing advantages—faster reaction times, lower perceived difficulty, and stronger attentional capture—relative to oblique or vertical alignments (Chen et al., 2019; Tsal, 1989). Yet, choice-based studies of context effects have not directly tied these perceptual benefits to the underlying attentional weights that determine preference construction. Thus, a direct behavioral test of whether spatial alignment affects the ease of TD vs. CD comparisons remains missing.

Experiment 4 addresses this gap by directly measuring the perceived difficulty of TD and CD comparisons under two alignment conditions: horizontal (linear) and oblique. By asking participants to rate difficulty in addition to making choices, we isolate attentional salience mechanisms and empirically test the key assumption that spatial alignment modulates attentional weights.

Unlike Experiment 1, where the main hypotheses concerned choice behavior—since dominance asymmetry was naturally defined in terms of accuracy—the present experiment focused on subjective difficulty ratings. In Experiment 4, we operationalized the attentional salience of each stimulus pair through the perceived difficulty of pairwise comparisons. This approach enabled us to quantify salience differences between Target-Decoy (TD) and Competitor-Decoy (CD) pairs in different alignments.

Hypotheses:

1. **Main effect of Pair Type:** TD comparisons will be rated as easier than CD comparisons.
2. **Main effect of Alignment:** Horizontal comparisons will be rated as easier than oblique comparisons, reflecting the horizontal attention advantage.
3. **Interaction:** The TD–CD ease advantage will be greatest when comparisons are horizontally aligned. This advantage should be attenuated when comparisons are presented obliquely; if spatial alignment systematically favors CD over TD, the TD–CD difference may be further reduced.

### 5.2 Method

#### 5.2.1 Participants

Forty-two university students (9 female) with normal or corrected-to-normal vision (M: 21.45 years, SD: 2.86, range: 17–27), participated in the experiment.

#### 5.2.2 Apparatus and stimuli

Experiment 4 was conducted on a 24-inch, 1920 × 1080-pixel monitor (16:9) viewed at approximately 60 cm, with all display calibration, rectangle-based stimulus generation, and attribute matching procedures identical to those described in Experiment 1. Stimuli were presented as black rectangles on a white background.

The spatial configuration of each stimulus pair followed the virtual equilateral triangle arrangement detailed for Experiments 2 and 3 (see Apparatus and Stimuli, Experiments 2-3). On each trial, two rectangles were simultaneously displayed at two of the three vertices of the triangle (left-top, right-top, or left-right combinations). The vertex coordinates, triangle base and height, and corresponding visual angles were as previously specified: base at *x* = 576 and *x* = 1344 (768 pixels/212.5 mm/20.1°), top at (*x* = 960, *y* = −125), triangle height 665 pixels (184.1 mm/17.4°). This ensured that spatial separation, size, and visual angle were strictly comparable to those in earlier experiments.

#### 5.2.3 Design

Experiment 4 employed a within-subjects forced-choice design in which participants selected the rectangle with the larger area from two simultaneously presented stimuli. Each trial consisted of either a Target-Decoy (TD) or a Competitor-Decoy (CD) pair, constructed from the corresponding triplets used in previous experiments.

For every combination of *Pair Type* (TD or CD) and *trial type* (range, frequency, range-frequency), four spatial presentation variants were used: *aligned0, aligned1, oblique0*, and *oblique1*. On each trial, only two of the three triangle vertices were occupied by rectangles. For CD pairs, aligned variants corresponded to the Competitor (C) and Decoy (D) occupying the left and right base vertices (aligned0: C left, D right; aligned1: D left, C right). Oblique variants corresponded to one item at a base vertex and the other at the top vertex (oblique0: C left, D top; oblique1: C right, D top). The same assignment logic was employed for TD pairs. For the final analysis, both aligned variants were combined into a single category, “aligned,” and both oblique variants were combined into “oblique.”

**Figure 7:**
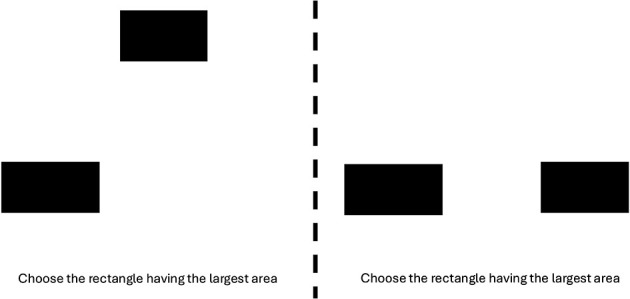
Example trials in Experiment 4. Stimuli pair are in oblique and horizontally aligned positions from left to right.

This design yielded 24 total experimental trials per participant, with presentation order fully randomized. Figure 7 displays two example trials. Each trial also included a difficulty rating task following the choice response. The number of trials was chosen to balance comprehensive coverage of all spatial arrangements with participant comfort, given the inclusion of rating tasks and the precision required for spatial positioning judgments.

#### 5.2.4 Procedure

At the start of each trial, a fixation cross appeared at the center of the screen for 500 ms. Immediately thereafter, two rectangle stimuli were displayed at the designated triangle vertices on a white background. The stimulus display remained visible until the participant made a choice response via key press; there was no imposed time limit for this response. Upon making their selection, participants proceeded to a rating task in which they rated the difficulty of their decision on a 7-point Likert scale, where one represented “extremely easy” and seven represented “extremely difficult”. The rating display also remained visible until a response was recorded via key press, with no time restriction. After each trial, a new fixation cross appeared for 500 ms before the onset of the next trial.

### 5.3 Results

Data from all forty-two participants were analyzed. We first applied the same duration exclusion criteria as preregistered in Experiment 1 (removing trials shorter than 100 ms or longer than 20 s), which resulted in the exclusion of 2.37% of trials.

**Figure 8:**
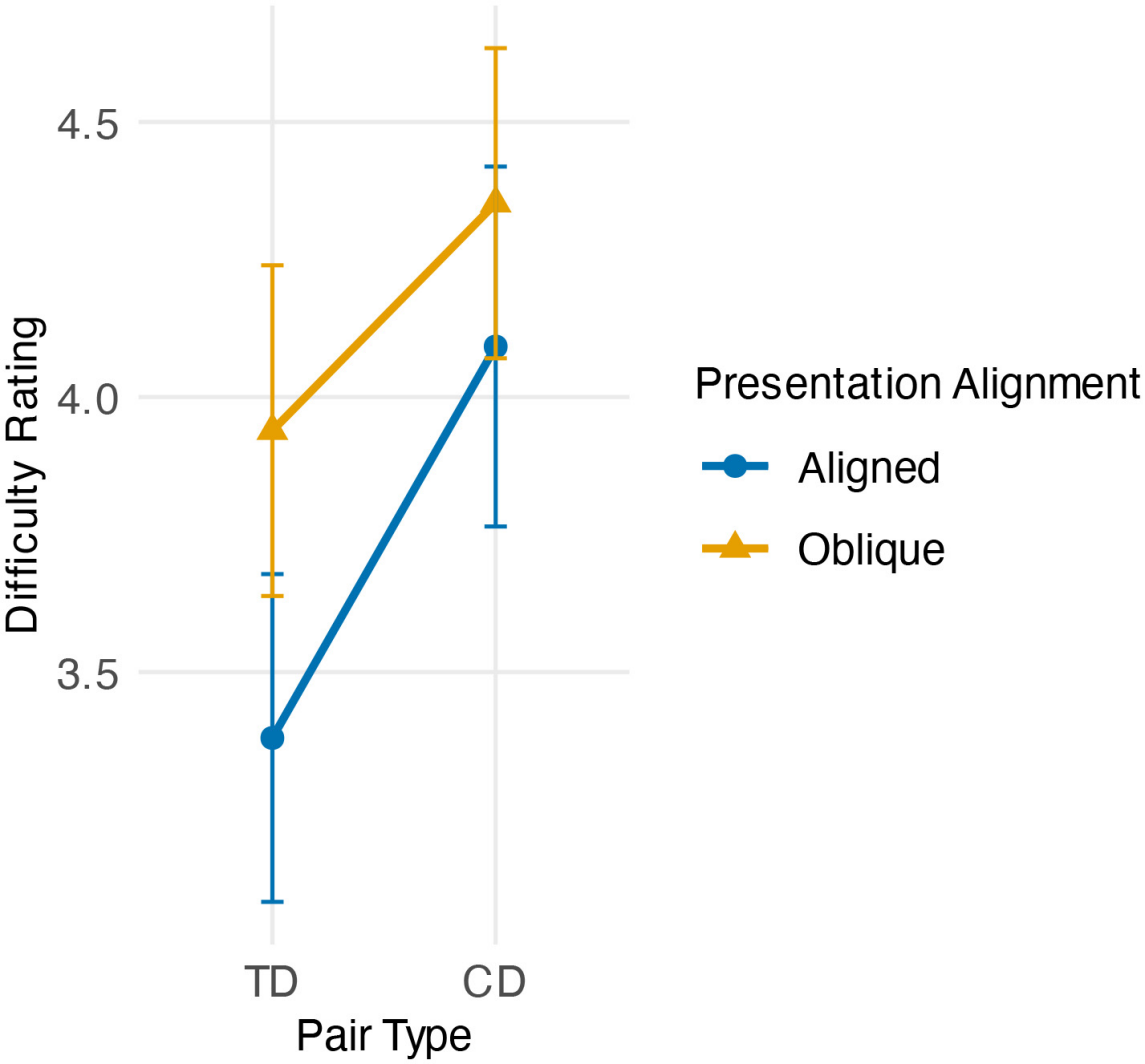
Mean difficulty ratings in Experiment 4 as a function of Pair Type (TD vs. CD) and Presentation Alignment (horizontal vs. oblique). Error bars indicate 95% confidence intervals of the mean (within-subject). The interaction reflects that the TD–CD ease advantage was more pronounced in the horizontal condition and attenuated in the oblique condition.

A two-way repeated-measures ANOVA with *Pair Type* (TD vs. CD) and *Presentation Alignment* (aligned vs. oblique) as within-subject factors was conducted on participants' difficulty ratings. The analysis revealed a main effect of Pair Type, with TD comparisons rated as easier than CD comparisons, *F*_(1, 41)_ = 27.22, *p* < 0.001, $\eta_{p}^{2}=0.40$. There was also a main effect of Presentation Alignment, with horizontal (aligned) comparisons rated as easier than oblique comparisons, *F*(1, 41) = 29.19, *p* < 0.001, $\eta_{p}^{2}=0.42$. Importantly, these effects were qualified by a significant interaction between Pair Type and Presentation Alignment, *F*_(1, 41)_ = 5.21, *p* = 0.028, $\eta_{p}^{2}=0.11$ (see Figure 8). To verify that the observed interaction was not driven by specific exclusion criteria, we conducted a sensitivity analysis similar to those employed in previous experiments, varying participant accuracy thresholds and trial-level RT cutoffs. As summarized in Supplementary Table S8, all main and interaction effects for our primary variable, difficulty rating, remained significant and stable across all tested parameter combinations.

Descriptive statistics are summarized in Table 3. CD comparisons were rated as more difficult (aligned: *M* = 4.09, *SD* = 1.05; oblique: *M* = 4.35, *SD* = 0.91) than TD comparisons (aligned: *M* = 3.38, *SD* = 0.96; oblique: *M* = 3.94, *SD* = 0.97).

**Table 3:** Mean difficulty ratings (*M*) and standard deviations (*SD*) by condition.

**Condition**	***M***	***SD***
CD aligned	4.09	1.05
CD oblique	4.35	0.91
TD aligned	3.38	0.96
TD oblique	3.94	0.97

To probe the interaction, we conducted four planned pairwise comparisons with Holm-Bonferroni correction (Table 4). TD–CD differences were significant in both the aligned [*t*_(41)_ = −4.99, *p*_holm_ < 0.001, *d*_*z*_ = −0.77] and oblique conditions [*t*_(41)_ = −3.87, *p*_holm_ < 0.001, *d*_*z*_ = −0.60]. The strongest difference was between TD-aligned and CD-oblique [*t*(41) = −6.95, *p*_holm_ < 0.001, *d*_*z*_ = −1.07]. By contrast, CD-aligned and TD-oblique did not differ reliably [*t*_(41)_ = 1.24, *p*_holm_ = 0.222, *d*_*z*_ = 0.19]. This pattern is consistent with our hypotheses: the TD–CD asymmetry advantage was maximal under horizontal alignment and attenuated under oblique alignment.

**Table 4:** Planned pairwise comparisons of difficulty ratings in Experiment 4.

**Contrast**	***t*_(41)_**	***p*_holm_**	***d*_*z*_**	**95% CI**
TD aligned vs. CD aligned	−4.99	< 0.001	−0.77	[−1.11, −0.42]
TD oblique vs. CD oblique	−3.87	< 0.001	−0.60	[−0.92, −0.26]
TD aligned vs. CD oblique	−6.95	< 0.001	−1.07	[−1.45, −0.69]
CD aligned vs. TD oblique	1.24	0.222	0.19	[−0.11, 0.50]

*p*-values are Holm-Bonferroni corrected.

Additionally, when accuracies were collapsed across presentation formats, no meaningful difference was observed between TD (M = 0.870, SD = 0.167) and CD (M = 0.850, SD = 0.145) conditions. A Wilcoxon signed-rank test indicated no significant difference in accuracy between TD and CD trials (*W* = 286, *p* = 0.277). A paired-samples *t*-test showed a consistent pattern, *t*_(41)_ = 1.16, *p* = 0.254, Cohen's *d* = 0.18. Further descriptive and inferential statistics for accuracy and reaction times are reported in Supplementary Section S7, Supplementary Tables S12–S14 for completeness.

### 5.4 Discussion

Unlike Experiment 1, in Experiment 4 the rectangles clearly lacked an item-wise asymmetric dominance of decoys in terms of choice accuracies. Beyond accuracy, Experiment 4 evaluated attentional biases as indexed by subjective difficulty ratings, which capture participants' relative ease of processing pairs under different spatial arrangements. It provided direct evidence that the accessibility of asymmetric dominance depends on spatial alignment. As predicted, TD comparisons were rated as easier than CD comparisons. We also observed a main effect of alignment, with horizontally aligned comparisons rated as easier than oblique ones, consistent with prior psychophysical work on horizontal processing advantages. Crucially, these two effects interacted: the TD–CD ease difference was strongest for horizontal pairs and attenuated for oblique pairs.

These findings help reconcile the puzzling discrepancy observed with rectangles. Rectangles, in Experiment 1, produced detectable pairwise asymmetry and produced robust AE in linear arrangements in prior studies, but they failed to yield AE in triangular displays (Experiment 3). Experiment 4 suggests a principled explanation: the weaker dominance asymmetry inherent to rectangles becomes accessible and behaviorally consequential only when supported by horizontal alignment. In triangular layouts, where TD and CD pairs are frequently oblique, this asymmetry is perceptually harder to access, undermining the conditions for AE to emerge. More broadly, our findings indicate that asymmetric dominance, as a condition, is also dependent on attentional weighing of pairwise comparisons.

## 6 General discussion

The present research set out to clarify the conditions under which the attraction effect (AE) emerges and to identify the mechanisms that drive it. By combining four experiments with reanalyses of prior work, we examined how item-based dominance asymmetries and presentation factors shape both pairwise judgments and multi-option choices. Our findings highlight the central role of asymmetric dominance as a boundary condition in producing AE, while also pointing to possible cognitive processes that manifest this boundary condition. In what follows, we summarize the main results, outline their theoretical implications, note methodological contributions, and discuss limitations and future directions.

### 6.1 Summary of findings

Across four experiments, we systematically investigated when and why the attraction effect (AE) emerges. A consistent pattern was observed: AE is closely tied to asymmetric dominance in pairwise comparisons. When stimuli such as stars produced strong dominance asymmetry of decoys, we observed both reliable asymmetries in pairwise judgments (Experiment 1) and robust AE in triplet choices (Experiment 2). By contrast, a reanalysis of prior work with bar stimuli (Spektor et al., 2022) showed no AE in triplets. Rectangles presented a more nuanced case: they produced clear asymmetry in pairwise comparisons (Experiment 1) and elicited AE in linear triplets in past studies, but failed to show AE in triangular arrangements (Experiment 3). Experiment 4 further clarified this discrepancy. Item-wise asymmetric dominance of decoys disappeared when rectangle pairs were presented in both horizontally aligned and oblique arrangements. At the same time, Experiment 4 showed that participants perceived target-decoy (TD) comparisons as easier than competitor-decoy (CD) comparisons overall, and that horizontal arrangements selectively enhance this perceived ease advantage. Together, these findings offered a coherent explanation for the mixed results involving rectangles.

### 6.2 Theoretical implications

Our findings have several important theoretical implications for understanding the attraction effect and decision-making processes more broadly. First, they provide strong support for the item-based definition of asymmetric dominance originally proposed by (Huber et al. 1982) as a prerequisite for the attraction effect. While much of the subsequent literature has adopted an attribute-based definition focused on specific attribute comparisons, our results suggest that the original, more general item-based conceptualization better captures the conditions necessary for producing the attraction effect. This account explains why stars consistently yield robust AE, while other stimuli, when they lack systematic dominance asymmetry, fail to produce it. In this sense, AE is best understood as a consequence of structural asymmetries, not as a universal artifact of decoy presence.

Second, our results help resolve apparent contradictions in the literature regarding the domain generality of the attraction effect. The failure to observe positive attraction effects in perceptual tasks with triangular arrangements (Spektor et al., 2018, 2021, 2022) had called into question whether the effect generalizes beyond higher-level decision domains. By demonstrating robust AE with perceptually complex stimuli (e.g., stars), our findings suggest that the attraction effect is indeed domain-general, but critically dependent on the presence of item-level dominance asymmetries.

Third, our item-based asymmetric dominance framework offers new insight into other established boundary conditions for the attraction effect. Previous work has demonstrated that attraction effects are attenuated or even abolished when attributes are commensurable–that is, when they can be directly compared on a common scale (Hayes et al., 2024; Banerjee et al., 2024b). Within our framework, commensurability likely facilitates holistic or integrated evaluation, wherein attributes are combined into a common currency or composite value before comparison. This process reduces the effective item-level dominance asymmetry of the decoy, as the decoy's attribute-wise advantage over the competitor may be offset by other attributes during integration. As a result, the key dominance-asymmetry boundary condition for robust attraction is weakened. Thus, our account suggests that the effects of commensurability arise primarily by suppressing dominance asymmetry, rather than via a separate cognitive process, unifying these phenomena within a single explanatory framework.

Furthermore, our dominance asymmetry framework offers a compelling explanation for the zero attraction effect observed by (Trendl et al. 2021) using naturalistic movie stimuli. Their study represents a particularly instructive case because it rigorously satisfied all boundary conditions identified by (Huber et al. 2014), yet still failed to demonstrate any attraction effect. (Trendl et al. 2021) concluded that “the attraction effect is limited to choice tasks where options are represented in a stylized format with objectively defined attributes,” but our findings suggest an alternative explanation grounded in the absence of genuine dominance asymmetry.

Crucially, while (Trendl et al. 2021) carefully constructed their stimuli to ensure that decoys were clearly dominated—with decoys rated at least three points lower than both targets and competitors on seven-point scales—they failed to establish the asymmetric dominance relationships that our work identifies as essential. Their experimental design explicitly created target-decoy pairs with “many shared genres that are likely to be perceived as similar” while ensuring “target-competitor pairs with no genre overlap that are likely to be perceived as different.” This design choice, while methodologically sound for establishing dominance, inadvertently created conditions for symmetric rather than asymmetric dominance.

Under our framework, the zero attraction effect in (Trendl et al. 2021)'s study reflects not the inherent limitations of naturalistic stimuli, but rather the absence of the critical boundary condition we identify: genuine item-level dominance asymmetry. Their decoys were symmetrically dominated by both targets and competitors, violating the fundamental requirement that decoys must create stronger dominance relationships with targets than with competitors. The fact that participants correctly identified and avoided decoys (choosing them in only 4.3% of trials) confirms that dominance was perceived; however, the symmetric nature of this dominance eliminated the differential comparison advantage that drives attraction effects.

This interpretation suggests that naturalistic stimuli can theoretically support attraction effects when dominance asymmetry conditions are met. The challenge lies in constructing naturalistic choice sets where decoys are more clearly dominated by targets than by competitors–a task that may require careful attention to attribute structure and similarity relationships that go beyond traditional experimental design considerations. (Trendl et al. 2021)'s findings thus provide strong convergent evidence for our theoretical framework: attraction effects emerge when decoys create asymmetric rather than merely symmetric dominance relationships, regardless of stimulus type or domain.

The case of rectangles further refined our theoretical understanding. While the presence or absence of dominance asymmetry in pairwise comparisons could explain the presence or absence of AE in rectangle triplets, at the process level, we found an interaction between the differential ease of comparison in specific item pairs (TD vs CD) and the presentation format. A speculative explanation is axis-congruence: because rectangles have width and height as defining attributes and are naturally encoded along orthogonal axes (in our case, horizontal and vertical axes), their evaluation may depend on whether these axes align with the spatial arrangement of options. When alignment is congruent (e.g., linear layouts), TD comparisons are facilitated, whereas incongruence (e.g., oblique triangular layouts) may reduce accessibility. This explanation is speculative and likely specific to rectangles or even bars, due to their attribute structures. Importantly, it remains an open question whether other visual perceptual stimuli would show similar susceptibility.

While we did not observe aggregate-level repulsion (reversed attraction) effects in any of our experiments, the existence of repulsion effects is robustly documented in the literature, either in the traditional regularity violation terms (Spektor et al., 2018, 2022; Banerjee et al., 2024a) or in broader operationalization–where instead of 3 alternative forced choice, a rank order of preference is recorded with decoys that are objectively superior and frequently chosen (Dumbalska et al., 2020). By carefully designed attribute structure and stimulus design, it may be possible to induce the negative attraction effect (still violating the regularity principle). Importantly, our theoretical framework extends to these phenomena: by incorporating attentional weights into the ordinal pairwise comparison model, the framework can account for how presentation format and context-dependent attention can shift the balance of subjective comparisons, thus explaining both standard attraction and its reversal—the repulsion effect—in certain choice contexts.

### 6.3 Methodological contributions

This study makes several methodological contributions to the field of decision-making research. We introduced a novel perceptual stimulus—the star-shaped figure—that effectively creates conditions for asymmetric dominance. This stimulus design offers researchers a new tool for investigating the attraction effect in perceptual domains while maintaining the critical feature of dominance asymmetry. The effectiveness of this stimulus derives from its complex perceptual properties that require integrating multiple visual features, thereby creating conditions where dominance relationships between alternatives become more pronounced.

Our methodological approach also demonstrates the importance of verifying dominance asymmetry at the pairwise level before implementing decoy paradigms. The underlying assumption that the process responsible for dominance asymmetry structure in pairwise comparisons would also operate in the triplet set to favor the target was supported by our findings. The design in Experiment 1, directly comparing accuracy in TD vs. CD pairs, provided a template for researchers to validate stimulus sets prior to testing AE in full triplet contexts. This validation step may be particularly valuable when working with perceptual stimuli where dominance relationships may be less intuitive than in value-based decision-making.

Additionally, by directly comparing different stimulus types (stars, bars, rectangles) within the same experimental paradigm, our experiments provide a controlled demonstration of how stimulus properties may influence dominance asymmetry and, in turn, the emergence of AE. This comparative approach could be extended to other types of stimuli to further map the boundary conditions of the effect.

Methodologically, our work also advances the literature by combining multiple behavioral measures: pairwise comparisons, triplet choices, and subjective difficulty ratings (Experiment 4). This triangulation allowed us to connect perceptual asymmetries observed in simple comparisons to the aggregate choice patterns observed in more complex sets, thereby providing converging evidence that AE is grounded in the psychological experience of comparative ease rather than being a statistical artifact of choice proportions.

Finally, we integrated new experimental evidence with reanalyses of existing datasets (e.g., Spektor et al., 2018). This combination of replication, extension, and synthesis increases confidence in the robustness of our conclusions while also clarifying why prior findings sometimes appeared inconsistent. Specifically, our results show that apparent contradictions are better understood as reflecting stimulus- and arrangement-specific boundary conditions, rather than a fundamental instability of AE itself.

### 6.4 Limitations and future directions

Despite its contributions, this work has several limitations that suggest important avenues for future research. First, while our novel star stimuli successfully produced dominance asymmetry and a reliable attraction effect, the specific properties that make these stimuli effective remain underspecified. A stronger dominance asymmetry could itself be a result of attribute incommensurability (Walasek and Brown, 2023; Hayes et al., 2024). Future research could systematically vary stimulus properties to identify which features are critical for generating effective asymmetric dominance in perceptual tasks.

Second, our findings with rectangles illustrate that attraction effects are not uniformly robust across stimulus types or presentation formats. The weaker but significant asymmetry of dominated decoys in horizontally presented pairwise comparisons vanished when pairs were distributed in horizontally aligned and oblique arrangements–a result that directly highlights the moderating role of spatial configuration in producing dominance asymmetry. This susceptibility of dominance asymmetry to stimulus presentation is consistent with evidence from prior work on the effect of presentation formats on AE, as reviewed by (Spektor et al. 2021), tested by (Cataldo and Cohen 2018, 2019); (Evans et al. 2019), and more recently by (Hasan et al. 2025). Whether this sensitivity of asymmetric dominance, and hence the attraction effect, to the presentation format generalizes to other perceptual stimuli beyond rectangles remains a fruitful direction for future research.

Third, although we inferred attentional weighting from choice and subjective difficulty ratings, we did not directly measure attentional allocation. Process-tracing approaches such as eye-tracking or mouse-tracking would provide more direct evidence about how participants allocate attention and engage in the comparison processes that drive the attraction effect. Previous research demonstrates that eye-tracking can illuminate the mechanisms underlying context effects in multiattribute choice, especially by revealing how attention is distributed between choice alternatives (Noguchi and Stewart, 2014; Marini et al., 2020; Molter et al., 2022; Cataldo and Cohen, 2019). For instance, (Noguchi and Stewart 2014) and (Marini et al. 2020) have shown that shifts in gaze patterns reflect comparative processes pivotal to the emergence (or suppression) of decoy effects, while (Molter et al. 2022) demonstrate that gaze-dependent evidence accumulation predicts behavioral responses in multi-alternative choice. Incorporating such measures in future work would not only clarify the attentional dynamics involved in perceptual attraction effects but would also help to distinguish between competing process models.

Fourth, the present work focused on pairwise asymmetric-dominance boundary conditions and presentation layout manipulations, but other situational constraints may also play a role. Factors such as decision time pressure, cognitive load could interact with the process responsible for dominance asymmetry, shaping the emergence and strength of AE. Prior research has demonstrated that time constraints may reduce the incidence or magnitude of context effects, including the attraction effect, by limiting the opportunity for deliberation and comparative processing (Spektor et al., 2021; Pettibone, 2012). Similarly, increased cognitive load might disrupt the necessary information integration, potentially attenuating or altering context effects [see, e.g., (Spektor et al. 2021)]. Examining the roles of these factors and their interactions would further refine the boundary conditions identified in this study.

Fifth, we did not include dedicated pairwise Target-Competitor (T-C) trials in Experiment 1 to empirically verify the assumed equivalence between core alternatives. Although T and C were designed to be equal on respective criterion values, this equivalence was achieved by design rather than confirmed behaviorally. Prior attraction effect research typically establishes such equivalence by design to ensure that any shift in relative choice share arises from the inclusion of the decoy rather than pre-existing bias between the core options. Future studies should explicitly incorporate T-C pairwise comparisons to validate this assumption and strengthen interpretations regarding asymmetric dominance and the robustness of the attraction effect under different stimulus types.

Yet, another limitation of the present work is that our two stimulus sets—rectangles and star-shaped figures—differ not only in their perceptual appearance but also in the specific judgment task associated with each. For rectangles, participants identified the item with the greatest area, whereas for stars, they judged the item requiring the least sand to complete a square. These differences raise the possibility that task-related demands or stimulus complexity may modulate the observed attraction effect. However, both stimulus sets were carefully designed such that variation was restricted to two orthogonal attributes, and the decoy manipulations were defined within the same attribute-based framework. We therefore interpret the present findings as evidence that the principle demonstrated here is not exclusive to one stimulus type or task, while recognizing that a broader range of stimuli and tasks will need to be examined in future work to establish the generality of the claims.

Seventh, the generalizability of attraction effects to naturalistic decision contexts remains an important open question (Frederick et al., 2014; Huber et al., 2014; Trendl et al., 2021). Our framework suggests that the absence of asymmetric dominance—rather than stimulus type itself—may explain null effects in naturalistic contexts. Future research should systematically construct naturalistic choice sets in which genuine item-level dominance asymmetry is clearly established, to determine whether attraction effects can emerge in ecologically valid decision environments and to further refine boundary conditions across diverse stimulus domains.

Eighth, our present findings are restricted to visual perceptual choice with controlled dominance asymmetry, leaving open the question of whether the attraction effect generalizes to other sensory modalities such as auditory or tactile domains. To guide this endeavor, the computational and neurocognitive framework articulated by (Busemeyer et al. 2019) is especially relevant: their sequential sampling models propose that context effects such as the attraction effect arise from dynamic processes involving shifting attentional focus, lateral inhibition among competing options, and real-time integration of attribute comparisons—core computations implemented through a distributed network of prefrontal and parietal regions. This framework predicts not only where in the brain such effects may emerge, but how their magnitude and neural signatures will depend on stimulus structure and deliberation time, offering specific, testable hypotheses for new domains. Although this account suggests the possibility of domain-general mechanisms for context effects (Pisauro et al., 2017; Levy and Glimcher, 2012; Philiastides et al., 2011), recent findings demonstrate modality-specific effects in the timing and locus of irrelevant information processing (Li et al., 2024). Thus, future studies must carefully design behavioral and neuroimaging paradigms tailored to each sensory modality, to empirically test whether dominance asymmetry produces attraction effects and whether their neural basis aligns with Busemeyer et al.'s process-level predictions across different domains.

A related and broader theoretical issue concerns the interpretation of attraction effects within the neuroscience of decision-making. Historically, the attraction effect has been viewed as a violation of rational value computation by economic models of choice. However, recent critiques (Hayden and Niv, 2021) contend that regions such as orbitofrontal cortex encode structured comparative signals rather than single value representations, suggesting that so-called “irrational” context effects may instead be lawful outcomes of the way brains process multi-attribute information. Our dominance asymmetry framework provides a bridge between these perspectives, proposing that attraction effects and preference reversals reflect structural features of comparative processing rather than failures of value encoding *per se*. Neuroimaging studies that rigorously manipulate dominance asymmetry, coupled with process-tracing of comparative attention and attribute integration, will be necessary to establish whether attraction effects are best explained by competitive structural mechanisms or truly signal limits to rational economic computation.

Together, these limitations and future directions highlight how specifying effective stimulus properties, testing the generalizability of dominance asymmetry beyond traditional visual domains, incorporating richer process-tracing and neuroimaging measures, and broadening the scope across sensory modalities, tasks, and theoretical frameworks can advance our understanding of when and why the attraction effect emerges. By integrating behavioral, computational, and neural approaches, future research will be positioned to clarify whether the attraction effect reflects deep structural mechanisms of comparative decision-making or signals fundamental constraints on value-based rationality.

### 6.5 Conclusion

Across four experiments, we resolved apparent contradictions in the literature by demonstrating that the attraction effect reliably emerges in perceptual decision-making when proper conditions of item-wise asymmetric dominance are met. These findings refine theoretical accounts of AE by highlighting the central role of pairwise comparison processes, while also providing methodological guidance for how to design and validate effective stimuli. More broadly, they support a unified framework in which the attraction effect reflects fundamental properties of human information processing that generalize from consumer choices to perceptual judgments. By establishing clear boundary conditions, our work clarifies when AE should and should not be expected, offering a stronger foundation for future investigations of this robust but sometimes elusive decision bias.

## Data Availability

The datasets presented in this study can be found in online repositories. The names of the repository/repositories and accession number(s) can be found below: https://osf.io/8yh9g.
