# Supplementary material for: The attraction effect in perceptual decision-making: a case of dominance asymmetry
Source: Front Psychol. 2025 Dec 18;16:1661748. doi: 10.3389/fpsyg.2025.1661748 (PMC12756130; doi:10.3389/fpsyg.2025.1661748)
Supplement: Supplementary file 1 [file Data_Sheet_1.pdf]

Supplementary Material

1 CRITERION VALUE COMPUTATION FOR STIMULI

Rectangle Stimuli

For rectangle stimuli, the criterion value was defined as the area:

$$\textit{Criterion value}_{\text{rectangle}} = w \times H$$

where w denotes the width and H the height of the rectangle. Both attributes were systematically varied to create the required ranges of target–decoy distances.

Quantitatively, the target–decoy distance (TD_dist%) for rectangles had a mean of 11.12% (SD = 2.13%), ranging from 7.23% to 14.54%.

Star Stimuli

1.0.0.1 Construction and Geometry

Star-shaped stimuli were derived from a base rectangle of width w and height H, with four inward-facing isosceles triangles (each of height d) removed—two from the vertical sides (base H, height d), two from the horizontal sides (base w, height d). This produces a star-like silhouette (see Supplementary Figure S1).

1.0.0.2 Criterion Value Formula (Stepwise Derivation)

The criterion value for the star equals the extra area needed to fill the star shape into a perfect square (of side H):

1. **Area of the square:**
$$A_{\text{square}} = H^2$$
2. **Area of the base rectangle:**
$$A_{\text{rect}} = w \times H$$
3. **Total area removed by triangles:**
$$A_{\text{removed}} = 2\left(\frac{1}{2}Hd\right) + 2\left(\frac{1}{2}wd\right) = Hd + wd$$
4. **Area of the star:**
$$A_{\text{star}} = wH - (Hd + wd) = wH - Hd - wd$$
5. **Criterion value:**
$$\text{CV}_{\text{star}} = H^2 - (wH - Hd - wd) = H^2 - wH + Hd + wd$$

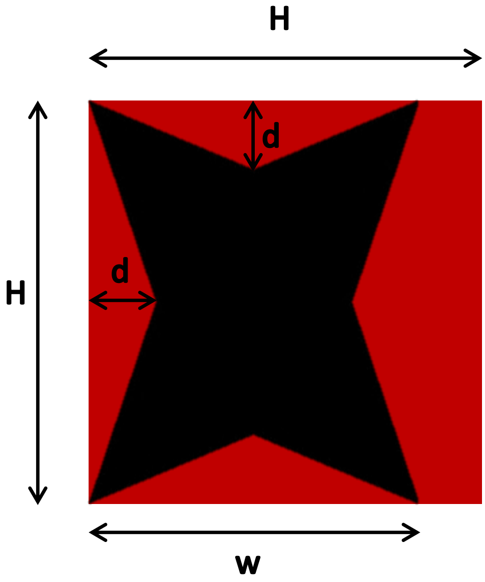

Figure S1. Example of a star-shaped stimulus derived from a rectangle (width w, height H) with four triangles (height d) removed. Figure is annotated to show w, H, d, and the extra area (criterion value) required to complete to a square. The filled-in square and removed triangles are depicted, with all parameters clearly labeled.

$$\boxed{\text{Criterion value}_{\text{star}} = H^2 - wH + Hd + wd}$$

Quantitatively, the target–decoy distance for stars (TD_dist%) had a mean of 13.97% (SD = 2.10%), ranging from 10.53% to 16.91%.

1.0.0.3 Practice Session: Comprehension of Criterion Value

A feedback-based practice session ensured participants understood the computation: participants could click star shapes to see them filled to a perfect square, with extra area highlighted and a numerical value displayed underneath. This provided intuitive feedback on the "extra sand" concept (criterion value). No special practice was needed for rectangles.

1.0.0.4 Comparability and Range

Both stimuli types are governed by two orthogonal, independently varied attributes. The stimulus file with all parameter values is available in the OSF repository.

2 SENSITIVITY ANALYSIS FOR EXPERIMENT 1

In attraction-effect paradigms, the *Target–Decoy distance percentage (TD_dist%)*—that is, the relative difference between the target and decoy on the relevant criterion dimension—should ideally have similar distributions across stimulus types to allow for a fair comparison of context effects. Matching TD_dist% ensures that any observed behavioral differences are not driven by differences in relative discriminability but instead reflect genuine psychological or perceptual effects.

In practice, however, perfect alignment of TD_dist% distributions is often difficult to achieve when stimuli differ geometrically or when parametric constraints interact nonlinearly, as in the case of the star stimuli. The nonlinear dependencies introduced by the star's criterion value make it infeasible to ensure an exact per-trial match with the rectangular stimuli without generating visually implausible or perceptually imbalanced shapes. Nevertheless, our goal was to create broadly comparable ranges across both stimulus types.

Upon closer inspection, we observed a modest but statistically significant difference in the TD_dist% distributions between rectangles ($M \approx 11.5\%$) and stars ($M \approx 14.0\%$) in our stimulus set (see Fig S2. This discrepancy arose from practical constraints in stimulus generation and was not intentional. Importantly, the chosen TD_dist% ranges are consistent with values commonly used in previous attraction-effect studies to manipulate discriminability (e.g., (Wedell, 1991; Farmer et al., 2017; Spektor et al., 2018))

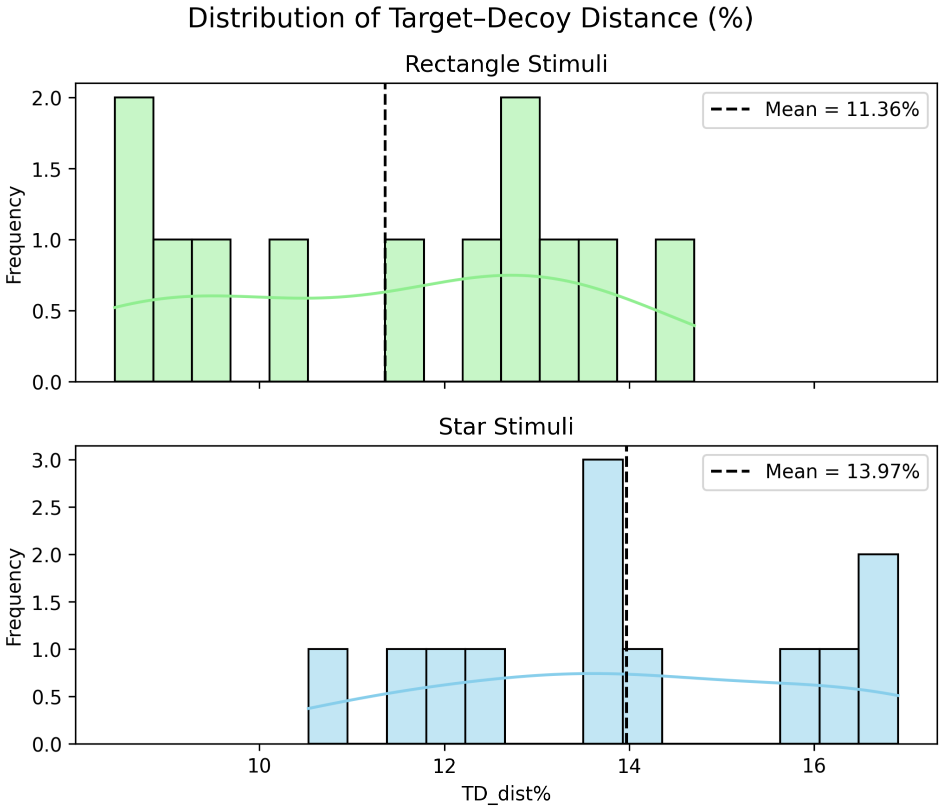

Figure S2. Distribution of target–decoy distance percentages ($\mathrm{TD_{dist}}\%$) for all rectangle and star stimuli included in the experiment. The distributions reveal a modest but statistically significant difference in the average TD_dist% between rectangles ($M \approx 11.5\%$) and stars ($M \approx 14.0\%$).

To directly address potential concerns that this difference might have influenced the main results, we conducted a *sensitivity analysis* restricted to the subset of trials for which the TD_dist% ranges overlapped for both rectangles and stars, calculated separately for each participant. While the original dataset comprised 2,817 trials, the filtered subset included 2,451 trials (approximately 87% of the total).

A Wilcoxon signed-rank test comparing the TD_dist% distributions across stimulus types revealed no significant difference after filtering (*W* = 790.00, *p* = 0.26). Crucially, the main and interaction effects for accuracy, reaction time, and difficulty ratings all remained robust and statistically significant, with effect sizes and significance levels nearly identical to those obtained from the full dataset (see Supplementary Table S1 and Figure S3).

Table S1. Repeated measures ANOVA results for accuracy, reaction time, and difficulty rating using only trials in the overlapping $\mathrm{TD}_{\mathrm{dist}}\%$ range for both rectangles and stars (sensitivity analysis). Each row reports the F value, p value, and partial eta squared (η_p^2) for the corresponding main effect or interaction.

**Factor**	$F(1,60)$	p	η_p^2
**Accuracy**			
Stimulus type	24.48	$< .001$	.29
Pair	79.39	$< .001$	.57
Stimulus type × Pair	7.73	.007	.11
**Reaction Time**			
Stimulus type	24.22	$< .001$	.29
Pair	8.07	.006	.12
Stimulus type × Pair	4.03	.049	.06
**Difficulty Rating**			
Stimulus type	3.70	.059	.06
Pair	19.27	$< .001$	.24
Stimulus type × Pair	6.96	.011	.10

Pairwise post hoc t-tests (sensitivity analysis): Using only trials in the overlapping $\mathrm{TD}_{\mathrm{dist}}\%$ range, the accuracy difference between pairs remained highly significant for the star stimuli ($t(60) = 7.13$, $p < .001$, Cohen's $d = 1.031$, mean difference = 0.181, SD = 0.199), mirroring the unfiltered analysis. For rectangle stimuli, the pairwise difference also remained robust and significant ($t(60) = 4.86$, $p < .001$, Cohen's $d = 0.659$, mean difference = 0.091, SD = 0.146).

These findings confirm that the reported effects are not artifacts of differences in TD_dist% distributions but instead reflect genuine underlying variations in attentional and perceptual processing between the two stimulus types.

3 BASELINE PREFERENCE CHECK IN EXPERIMENT 1

To ensure that the observed dominance asymmetry in Experiment 1 was not confounded by a baseline preference for either the wide (W) or narrow (N) core option, we conducted a generalized linear mixed-effects model (GLMM) analysis. The model predicted trial-level accuracy (correct/incorrect) with fixed effects of pair type (target-decoy [TD] vs. competitor-decoy [CD]), target type (W vs. N), their interaction, and stimulus type (rectangle vs. star). A random intercept for participant was included to account for repeated measures.

3.1 Model specification

The GLMM was fitted using the binomial family with a logit link. The formula in `R` notation was:

```
correct ~ pair * target_type + stimulus_type + (1 | userId)
```

where `correct` is a binary variable (1 = correct, 0 = incorrect), `pair` is TD or CD, `target_type` is W or N, `stimulus_type` is rectangle or star, and `userId` is the participant identifier.

The analysis included 2,809 trials from 61 participants. The random effect variance for participant intercepts was 0.45 (SD = 0.67). Model fit indices were AIC = 2,333.8 and BIC = 2,369.4.

3.2 Results

Table S2. Fixed effects estimates from the GLMM predicting accuracy in Experiment 1. Odds ratios (OR) are derived from the logistic model coefficients.

Predictor	Estimate	*SE*	*z*	*p*	OR
(Intercept)	1.67	0.14	11.56	$< .001$	5.31
Pair TD	1.15	0.16	7.10	$< .001$	3.15
Target type W	-0.01	0.13	-0.08	.94	0.99
Stimulus type star	-0.66	0.11	-6.06	$< .001$	0.52
Pair TD × Target type W	-0.14	0.22	-0.63	.53	0.87

Note. SE = standard error; OR = odds ratio.

As shown in Table S2, the main effect of pair type was significant, with higher accuracy for TD pairs than for CD pairs (odds ratio = 3.15). There was no significant main effect of target type (W vs. N; $p = .94$), indicating that participants did not exhibit an inherent bias toward either core option. The interaction between pair type and target type was also nonsignificant ($p = .53$), suggesting that the accuracy advantage for TD pairs was consistent regardless of target type.

These results confirm that the dominance asymmetry observed in Experiment 1 cannot be attributed to a baseline preference for either the wide or narrow core option. The effect of pair type on accuracy was robust and stable across target conditions, reinforcing the validity of the main experimental findings.

4 PILOT STUDY AND STIMULUS ADJUSTMENT PROCEDURE IN EXPERIMENT 2

We conducted a pilot study prior to Experiment 2, employing triplet sets typical of attraction effect designs (Target, Competitor, Decoy) with attributes systematically varied. The pilot revealed a moderate bias in favor of one core option (typically the wider star) in the final triplet choice.

In Experiment 1, our design focused exclusively on pairwise Target–Decoy (TD) and Competitor–Decoy (CD) comparisons, omitting dedicated Target–Competitor (T–C) trials. While core options were carefully designed to be equivalent on the relevant criterion dimension (see Section 6.4, Limitations and Future Directions, main text), formal behavioral verification of T–C equivalence was not performed. Although our GLMM analyses found no significant target type effects under pairwise conditions in Experiment 1, the pilot study suggested that triplet presentations might amplify even subtle preference tendencies, necessitating empirical calibration in Experiment 2.

To ensure that such a bias did not confound the measurement of the attraction effect or reduce the effect sizes, we implemented a targeted stimulus adjustment. Specifically, the width of the narrower star shape (w_2) was increased by 10 pixels for Experiment 2. This correction was calibrated to restore choice parity between core shapes within triplet sets, supporting the integrity of the subsequent analyses.

A graphical depiction of these adjustments is presented in Supplementary Figure S4. It illustrates the core star stimuli in 2D attribute space, visually depicting the 10-pixel increase in w_2 applied for Experiment 2. The figure provides a clear visual comparison of the core item configurations across experimental designs.

In summary, our pilot-based adjustments and rigorous baseline checks across designs strengthen the validity and interpretability of context-dependent choice effects, while also highlighting the importance of direct core alternative comparisons in future studies.

5 REPLICATION OF SPEKTOR ET AL. (2018) AND REANALYSIS OF SPEKTOR ET AL. (2022)

Rationale for Replication

In our study, the central thesis is that asymmetric dominance of the decoy is a necessary precondition for the emergence of the attraction effect in triplet choice sets. Given that our Experiment 1 revealed only weak asymmetric dominance of decoys for rectangles in pairwise comparisons, we predicted that such stimuli should not generate a robust repulsion effect, but rather a reduced or null attraction effect.

Interestingly, the same rectangular stimuli have been shown to produce both positive attraction effects—when arranged in a linear display layout (as in Trueblood et al. (2013) and Experiment 4a in Spektor et al. (2018))—and negative effects, as reported in Experiments 1, 2, 3, and 4b of Spektor et al. (2018) when presented in a triangular display layout. Moreover, other perceptual stimuli, such as bars, have also produced negative attraction (repulsion) effects in triangular layouts (Spektor et al., 2022).

Our analysis of both Spektor et al. (2018) and Spektor et al. (2022) revealed a methodological feature uncommon among attraction effect studies. Typically, such studies equate the two core alternatives on the decision-relevant dimension or criterion value (e.g., equal area, equal value-for-money), ensuring that any bias in choice behavior can be attributed solely to the presence of a decoy. In contrast, these two studies implemented a richer within-subject design with multiple, simultaneously manipulated factors—including correct option/larger, set type, difficulty, target, decoy type, and attribute distance. The stimuli consisted of either rectangle or bar pairs (wide-but-low and narrow-but-high), with participants asked to select the option with the highest total criterion (area or total filled length).

Crucially, the experimental designs in both studies manipulated the discriminability between core options (e.g., 3% versus 7% difference), a feature rarely included but theoretically important for understanding the strength of pre-decoy preferences.

Because the data from both studies are publicly available, it is feasible to reanalyse them to assess whether the originally reported negative attraction effects persist when all manipulations are explicitly modelled. However, given the likelihood that these manipulations interact with the underlying requirement of asymmetric dominance for the decoy, we chose instead to conduct a conceptual replication of Spektor et al. (2018)'s triangular triplet design using stimuli of comparable size.

Nonetheless, we did reanalyze the data from Spektor et al. (2022) and found that the previously reported reversed attraction effect is not robust, but rather better explained as null findings when multiple design factors are jointly modelled.

Model Specification for Reanalysis

We fitted a series of generalized linear mixed-effects models (GLMMs) using the lme4 package (Bates et al., 2015), based on data from 54,916 trials across 55 participants. The models predicted binary responses (NH = 1, WL = 0) and included random intercepts for participants. The primary predictor of interest was the *target* variable, which indicates which of the two core options (NH or WL) was designated as the target on a given trial. This variable captures the hypothesized influence of the decoy.

Candidate models included progressively more complex specifications, beginning with a null model (random intercepts only), an additive model including *larger*, *target*, *difficulty*, and *condition*, and extending to interaction models incorporating two-way and three-way interactions among these predictors. In total, fifteen models were estimated, labeled A through D3 in Table S3.

**Model**	**Formula**
A0	True_response_bin ∼ 1 + (1|subject_id)
A	True_response_bin ∼ larger + target + difficulty + condition + (1|subject_id)
B1	True_response_bin ∼ larger ∗ target + difficulty + condition + (1|subject_id)
B2	True_response_bin ∼ larger ∗ difficulty + target + condition + (1|subject_id)
B2_reduced	True_response_bin ∼ larger ∗ difficulty + target + (1|subject_id)
B3	True_response_bin ∼ larger ∗ condition + target + difficulty + (1|subject_id)
B4	True_response_bin ∼ target ∗ difficulty + larger + condition + (1|subject_id)
B5	True_response_bin ∼ target ∗ condition + larger + difficulty + (1|subject_id)
B6	True_response_bin ∼ difficulty ∗ condition + larger + target + (1|subject_id)
C1	True_response_bin ∼ larger ∗ target + larger ∗ difficulty + condition + (1|subject_id)
C2	True_response_bin ∼ larger ∗ difficulty + condition ∗ target + (1|subject_id)
C3	True_response_bin ∼ larger ∗ condition + difficulty ∗ target + (1|subject_id)
D1	True_response_bin ∼ larger ∗ difficulty ∗ condition + target + (1|subject_id)
D2	True_response_bin ∼ larger ∗ target ∗ condition + difficulty + (1|subject_id)
D3	True_response_bin ∼ larger ∗ target ∗ difficulty + condition + (1|subject_id)

Table S3. Candidate GLMER models for binary response (`True_response_bin`), from null (A0) to additive (A) to interactions (B1–B6, C1–C3, D1–D3).

The best-fitting model (Model D1) can be expressed as:

$$\text{logit}\big(P(\text{NH response})\big) = \beta_0 + \beta_1\,\text{larger} + \beta_2\,\text{difficulty} + \beta_3\,\text{condition} + \beta_4\,\text{target} + \beta_{5-8}\,\text{interactions} + u_{\text{subject}}. \tag{S1}$$

Expanded form (on the logit scale):

$$\begin{aligned}\text{logit}\big(P(Y_{ij}=1)\big) &= \beta_0 + \beta_1\,\text{larger}_{ij} + \beta_2\,\text{difficulty}_{ij} + \beta_3\,\text{condition}_{ij} + \beta_4\,\text{target}_{ij} \\ &\quad + \beta_5\,(\text{larger}\times\text{difficulty})_{ij} + \beta_6\,(\text{larger}\times\text{condition})_{ij} + \beta_7\,(\text{difficulty}\times\text{condition})_{ij} \\ &\quad + \beta_8\,(\text{larger}\times\text{difficulty}\times\text{condition})_{ij} + u_{0j}.\end{aligned} \tag{S2}$$

Where:

- Y_{ij}: binary response for trial i from participant j, coded as 1 = NH chosen, 0 = WL chosen.

- $\text{logit}(p) = \ln\left(\frac{p}{1-p}\right)$: log-odds link function for binomial models.
- β_0: fixed intercept (baseline log-odds when predictors are at their reference levels).
- $\beta_1, \ldots, \beta_4$: main effects of *larger* (which option had greater fill), *difficulty* (3% vs. 7%), *condition* (baseline vs. preferential), and *target*.
- $\beta_5, \beta_6, \beta_7$: coefficients for two-way interactions.
- β_8: coefficient for the three-way interaction.
- $u_{0j} \sim \mathcal{N}(0, \sigma^2)$: subject-specific random intercept, capturing individual differences in baseline choice tendencies.

In `R` notation (using `lme4`; Bates et al. (2015)), the model was specified as:

```
True_response_bin ~ larger * difficulty * condition
                        + target + (1 | subject_id)
```

Here, `larger * difficulty * condition` expands into all main effects and their interactions up to the three-way term, `+ target` adds the fixed effect of the decoy-targeted option, and `(1 | subject_id)` specifies random intercepts per participant.

Model Comparison

Table S4 reports the AIC and BIC values for all fifteen models. Both information criteria converged in identifying Model D1 (highlighted in the table) as the preferred specification.

Table S4. Model comparison based on AIC and BIC values. The best-fitting model (D1) is highlighted.

Model	AIC	BIC
Null	74771.88	74789.70
A	61746.59	61800.07
B1	61747.69	61810.08
B2	61261.99	61324.38
B2_reduced	61278.17	61331.65
B3	61683.93	61746.32
B4	61746.17	61808.56
B5	61743.18	61805.57
B6	61748.57	61810.97
C1	61262.81	61334.12
C2	61258.78	61330.09
C3	61683.57	61754.88
D1	61187.11	61276.24
D2	61684.65	61773.78
D3	61266.01	61355.15

Results

In the best-fitting model, the fixed-effect estimate for *target* was small and not statistically significant ($\beta = 0.0335$, $p = 0.09$). By contrast, *larger*, *difficulty*, and their higher-order interactions produced reliable effects. These findings indicate that the *target* variable—central to the definition of the attraction effect—did not substantially influence choice once other design manipulations were accounted for.

This conclusion is illustrated in Figure S5, which displays the fixed-effect estimates from the best-fitting model (D1) with 95% confidence intervals. The plot shows reliable effects of *larger*, *difficulty*, and their

interactions, whereas the coefficient for *target* is small and its confidence interval overlaps zero, indicating no substantial influence of the decoy-designated option.

5.1 Conclusion

The re-analysis indicates that the reversed attraction effect reported by Spektor et al. (2022) is not robust once multiple design factors are simultaneously modeled. The effect of the *target* variable is best characterized as null, consistent with the absence of a reliable attraction—or repulsion—effect under bar stimuli.

6 ROBUSTNESS OF RESULTS ACROSS EXPERIMENTS

To assess the robustness of our findings, we systematically examined the effects of commonly used participant- and trial-level exclusion criteria on the primary measure of each experiment. Experiment 1 was pre-registered, providing a reference for exclusion thresholds, whereas other experiments were not. Thresholds were therefore selected based on Experiment 1 and previous literature, including the one replicated in Experiment 3 (Spektor et al., 2018).

Exclusion Criteria

Participant-level exclusion was based on catch trial accuracy. Two thresholds were considered: 0.8 (reflecting the pre-registered criterion in Experiment 1) and a more lenient 0.677, informed by prior literature (Spektor et al., 2018). Participants performing below the threshold were excluded.

Trial-level exclusion removed implausibly fast or slow responses. Trials with reaction times below 100 ms were excluded. Upper bounds included three alternatives: a fixed 20,000 ms threshold (pre-registered in Experiment 1), a fixed 8,000 ms threshold (used in prior literature), and a participant-specific threshold defined as the 75th percentile plus 1.75 times the interquartile range of each participant's RT distribution.

Robustness Analysis Approach

For each experiment and each combination of participant- and trial-level exclusion criteria, we recomputed the primary measure (relative share of target, RST, or other experiment-specific dependent variables). Frequentist analyses used one-sample t-tests against null values (0.5 for RST), with effect sizes calculated as Cohen's d. Bayesian analyses were conducted in R using the `BayesFactor` package to compute Bayes factors. Experiment 4 did not include catch trials.

Results

Across all four experiments, the direction and statistical significance of the primary effects were preserved under all exclusion criteria.

Tables S5–S8 provide detailed results for each experiment, including sample sizes, trials retained, mean and SD of primary measures, t-values, p-values, effect sizes, and Bayes factors. Each table follows a similar layout.

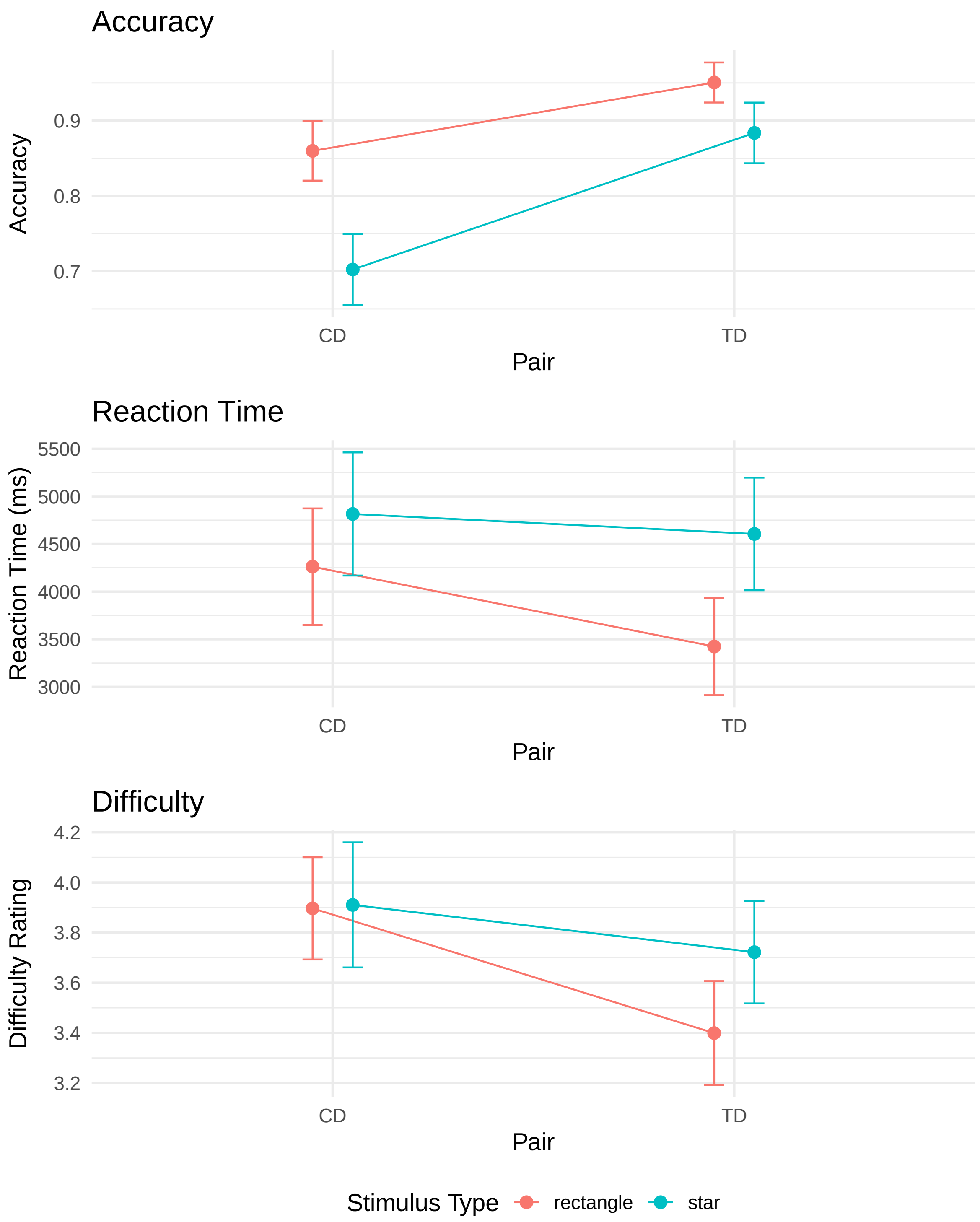

Figure S3. Interaction effects of stimulus type and pair on accuracy, reaction time, and difficulty rating, using only trials with matched overlapping $\mathrm{TD}_{\mathrm{dist}}\%$ ranges between rectangles and stars (sensitivity analysis). Error bars represent 95% within-subject confidence intervals.

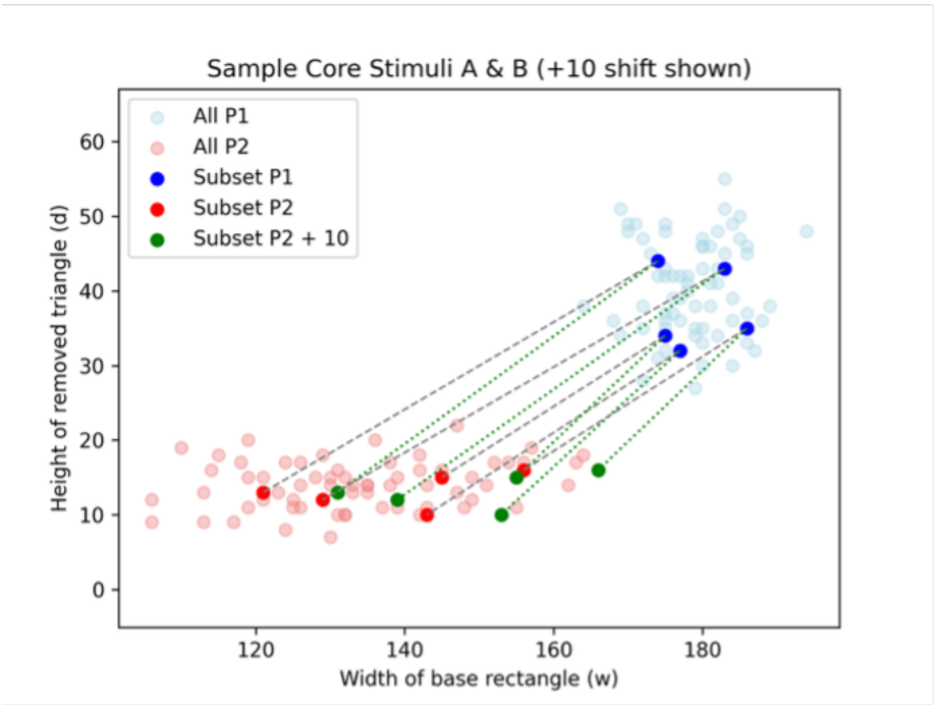

Figure S4. Core star items plotted in 2D attribute space. The adjustment of the narrower (w_2) item by 10 pixels for Experiment 2 is indicated, highlighting the bias correction relative to Experiment 1.

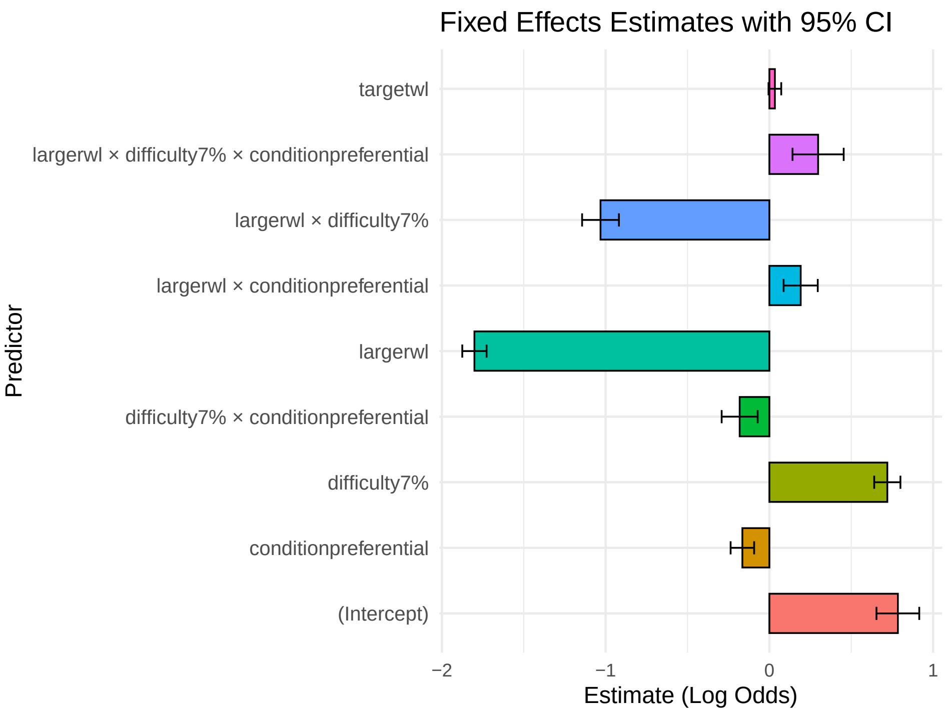

Figure S5. Estimated effects of each predictor on the probability of choosing the narrow–high (NH) option. Predictors are listed vertically; the horizontal axis shows the size and direction of each effect on the log-odds scale. Error bars are 95% confidence intervals. Effects to the right (positive) increase the chance of choosing NH; effects to the left (negative) decrease it.

Table S5. Robustness of Experiment 1 results across different participant- and trial-level exclusion criteria. **part_threshold:** participant-level catch accuracy threshold; **RT_Low / RT_Upper:** lower and upper bounds for reaction times (ms); **n_users / n_trials:** number of participants and trials before exclusion; **Trials removed%:** proportion of trials removed due to RT criteria; **effect / F / num_df / den_df / p_value / pes:** effect tested, F statistic, numerator and denominator degrees of freedom, p-value, and partial eta squared.

part_threshold	RT Low	RT Upper	n_users	n_trials	Trials removed%	effect	F	num_df	den_df	p	pes
0.8	100	20000	61	2809	0.041	stimulus_type	11.06	1	60	0.002	0.156
0.8	100	20000	61	2809	0.041	pair	75.82	1	60	< 0.001	0.558
0.8	100	20000	61	2809	0.041	stimulus_type:pair	9.81	1	60	0.003	0.140
0.8	100	15000	61	2745	0.063	stimulus_type	10.60	1	60	0.002	0.150
0.8	100	15000	61	2745	0.063	pair	64.35	1	60	< 0.001	0.517
0.8	100	15000	61	2745	0.063	stimulus_type:pair	9.15	1	60	0.004	0.132
0.8	100	10000	61	2567	0.123	stimulus_type	10.71	1	60	0.002	0.151
0.8	100	10000	61	2567	0.123	pair	56.12	1	60	< 0.001	0.483
0.8	100	10000	61	2567	0.123	stimulus_type:pair	9.44	1	60	0.003	0.136
0.8	100	8000	61	2421	0.173	stimulus_type	11.78	1	60	0.001	0.164
0.8	100	8000	61	2421	0.173	pair	51.52	1	60	< 0.001	0.462
0.8	100	8000	61	2421	0.173	stimulus_type:pair	11.41	1	60	0.001	0.160
0.677	100	20000	61	2809	0.041	stimulus_type	11.06	1	60	0.002	0.156
0.677	100	20000	61	2809	0.041	pair	75.82	1	60	< 0.001	0.558
0.677	100	20000	61	2809	0.041	stimulus_type:pair	9.81	1	60	0.003	0.140
0.677	100	15000	61	2745	0.063	stimulus_type	10.60	1	60	0.002	0.150
0.677	100	15000	61	2745	0.063	pair	64.35	1	60	< 0.001	0.517
0.677	100	15000	61	2745	0.063	stimulus_type:pair	9.15	1	60	0.004	0.132
0.677	100	10000	61	2567	0.123	stimulus_type	10.71	1	60	0.002	0.151
0.677	100	10000	61	2567	0.123	pair	56.12	1	60	< 0.001	0.483
0.677	100	10000	61	2567	0.123	stimulus_type:pair	9.44	1	60	0.003	0.136
0.677	100	8000	61	2421	0.173	stimulus_type	11.78	1	60	0.001	0.164
0.677	100	8000	61	2421	0.173	pair	51.52	1	60	< 0.001	0.462
0.677	100	8000	61	2421	0.173	stimulus_type:pair	11.41	1	60	0.001	0.160

Table S6. Robustness of Experiment 2 results across different participant- and trial-level exclusion criteria. **CatchAcc_Thres:** catch trial accuracy threshold for participant exclusion; **RT_Lower_ms / RT_Upper_ms:** lower and upper bounds for reaction times; **PSpec:** a participant-specific upper threshold, defined as the 75th percentile plus 1.75 times the interquartile range of that participant's RT distribution. **InitN / InitTrials:** initial participants and trials; **Part_N / Trials_after_Part:** after participant-level exclusion; **Final_N / Final_Trials:** after both exclusions; **Trials_Removed_%:** percentage of trials removed; **Mean_RST / Std_RST:** mean and SD of relative share of target; **t_stat / p_val / Cohen's d / BF10:** t-test statistics, p-values, effect size, and Bayes factor.

Condition	CatchAcc	RT Lower (ms)	RT Upper (ms)	Init N	Init Trials	Part N	Trials after Part	Final N	Final Trials	Trials Removed %	Mean RST	Std RST	t	p	Cohen's d	BF10
No Tech-Error Exclusion	0.80	100	20000	54	9720	52	9360	52	9048	3.33	0.54	0.05	5.73	<0.001	0.80	29268.82
No Tech-Error Exclusion	0.80	100	8000	54	9720	52	9360	52	8044	14.06	0.54	0.05	5.73	<0.001	0.79	28772.98
No Tech-Error Exclusion	0.80	100	PSpec	54	9720	52	9360	52	8832	5.64	0.54	0.05	5.60	<0.001	0.78	18659.41
No Tech-Error Exclusion	0.68	100	20000	54	9720	52	9360	52	9048	3.33	0.54	0.05	5.73	<0.001	0.80	29268.82
No Tech-Error Exclusion	0.68	100	8000	54	9720	52	9360	52	8044	14.06	0.54	0.05	5.73	<0.001	0.79	28772.98
No Tech-Error Exclusion	0.68	100	PSpec	54	9720	52	9360	52	8832	5.64	0.54	0.05	5.60	<0.001	0.78	18659.41
With Tech-Error Exclusion	0.80	100	20000	43	7740	42	7560	42	7252	4.07	0.54	0.05	4.82	<0.001	0.74	1035.96
With Tech-Error Exclusion	0.80	100	8000	43	7740	42	7560	42	6269	17.08	0.54	0.05	4.86	<0.001	0.75	1176.81
With Tech-Error Exclusion	0.80	100	PSpec	43	7740	42	7560	42	7038	6.91	0.54	0.05	4.68	<0.001	0.72	702.82
With Tech-Error Exclusion	0.68	100	20000	43	7740	42	7560	42	7252	4.07	0.54	0.05	4.82	<0.001	0.74	1035.96
With Tech-Error Exclusion	0.68	100	8000	43	7740	42	7560	42	6269	17.08	0.54	0.05	4.86	<0.001	0.75	1176.81
With Tech-Error Exclusion	0.68	100	PSpec	43	7740	42	7560	42	7038	6.91	0.54	0.05	4.68	<0.001	0.72	702.82

Table S7. Robustness of Experiment 3 results across different participant- and trial-level exclusion criteria. **CatchAcc_Thres:** catch trial accuracy threshold for participant exclusion; **RT_Lower_ms / RT_Upper_ms:** lower and upper bounds for reaction times (ms); **InitialN / InitialTrials:** number of participants and trials before exclusion; **Part_N / Trials_after_Part / Final_N / Final_Trials:** number of participants and trials after exclusions; **Trials_Removed_%:** percentage of trials removed; **Mean_RST / Std_RST:** mean and SD of relative share of target; **t_stat / p_val / Cohen's d / BF10:** t-test statistic, p-value, effect size, and Bayes factor.

Condition	CatchAcc	RT Lower (ms)	RT Upper (ms)	Initial N	Initial Trials	Part N	Trials after Part	Final N	Final Trials	Trials Removed %	Mean RST	Std RST	t	p	Cohen's d	BF10
No Tech-Error Exclusion	0.8	100	20000	76	12314	70	11342	70	11216	1.11	0.50	0.05	0.09	0.93	0.01	0.13
No Tech-Error Exclusion	0.8	100	8000	76	12314	70	11342	70	10889	3.99	0.50	0.05	0.01	0.99	0.00	0.13
No Tech-Error Exclusion	0.8	100	PSpec	76	12314	70	11342	70	10694	5.71	0.50	0.05	0.29	0.78	0.03	0.14
No Tech-Error Exclusion	0.677	100	20000	76	12314	72	11666	72	11537	1.11	0.50	0.05	0.41	0.68	0.05	0.14
No Tech-Error Exclusion	0.677	100	8000	76	12314	72	11666	72	11193	4.06	0.50	0.05	0.34	0.73	0.04	0.14
No Tech-Error Exclusion	0.677	100	PSpec	76	12314	72	11666	72	10998	5.73	0.50	0.05	0.58	0.56	0.07	0.15
With Tech-Error Exclusion	0.8	100	20000	74	11990	68	11018	68	10892	1.14	0.50	0.05	0.03	0.98	0.00	0.13
With Tech-Error Exclusion	0.8	100	8000	74	11990	68	11018	68	10566	4.10	0.50	0.05	-0.05	0.96	-0.01	0.13
With Tech-Error Exclusion	0.8	100	PSpec	74	11990	68	11018	68	10371	5.87	0.50	0.05	0.22	0.82	0.03	0.14
With Tech-Error Exclusion	0.677	100	20000	74	11990	70	11342	70	11213	1.14	0.50	0.05	0.36	0.72	0.04	0.14
With Tech-Error Exclusion	0.677	100	8000	74	11990	70	11342	70	10870	4.16	0.50	0.06	0.28	0.78	0.03	0.14
With Tech-Error Exclusion	0.677	100	PSpec	74	11990	70	11342	70	10675	5.88	0.50	0.06	0.52	0.60	0.06	0.15

Table S8. Robustness of Experiment 4 difficulty ratings across different trial-level RT exclusion criteria. **RT_Low / RT_Upper:** lower and upper bounds for reaction times (ms); **n_users_retained / n_users_removed:** participants included / excluded; **n_trials_retained / RemovedTrials%:** trials kept and percentage removed; **effect / F / num_df / den_df / p_value / pes:** effect tested, F statistic, numerator and denominator degrees of freedom, p-value, and partial eta squared.

RT Low	RT Upper	n_users retained	n_users removed	n_trials retained	RemovedTrials%	effect	F	num_df	den_df	p	pes
100	20000	42	0	989	2.37	pair	27.22	1	41	<0.001	0.399
100	20000	42	0	989	2.37	presentation_format	29.19	1	41	<0.001	0.416
100	20000	42	0	989	2.37	pair:presentation_format	5.21	1	41	0.028	0.113
100	15000	42	0	969	4.34	pair	26.44	1	41	<0.001	0.392
100	15000	42	0	969	4.34	presentation_format	28.77	1	41	<0.001	0.412
100	15000	42	0	969	4.34	pair:presentation_format	6.36	1	41	0.016	0.134
100	10000	42	0	929	8.29	pair	23.50	1	41	<0.001	0.364
100	10000	42	0	929	8.29	presentation_format	27.52	1	41	<0.001	0.402
100	10000	42	0	929	8.29	pair:presentation_format	7.39	1	41	0.010	0.153
100	8000	42	0	903	10.86	pair	23.12	1	41	<0.001	0.361
100	8000	42	0	903	10.86	presentation_format	27.75	1	41	<0.001	0.404
100	8000	42	0	903	10.86	pair:presentation_format	6.54	1	41	0.014	0.138

7 SUPPLEMENTARY RESULTS: DESCRIPTIVE AND SECONDARY ANALYSES ACROSS EXPERIMENTS

In this section, we report descriptive statistics and secondary inferential analyses for all experiments. All primary analyses are reported in the main text; here, we provide detailed participant-wise summaries and additional exploratory comparisons to support transparency and interpretability.

For each experiment, descriptive statistics include the mean, standard deviation (SD), median, interquartile range (IQR), and 95% confidence intervals (CI) for each dependent variable (accuracy, reaction time, and difficulty ratings), separately for each combination of experimental conditions (e.g., stimulus type $\times$ pair type). The number of participants (N) retained reflects exclusion of low-performing individuals (catch trial accuracy < 0.8) and trials outside the duration cutoffs ($<$100 ms or $>$20,000 ms). These statistics provide an overview of central tendency, variability, and precision of estimates across conditions.

Where applicable, secondary inferential analyses are also reported. These include repeated-measures ANOVAs and post-hoc paired comparisons, conducted to explore patterns not directly addressed in the primary analyses. Significant effects of experimental manipulations are summarized with standard APA-style statistics (F, df, p, partial η^2 or t, df, p, Cohen's d). These supplementary analyses are intended to provide additional context to the main findings and to allow readers to evaluate the robustness and consistency of the observed effects across experiments.

Tables S9–S12 present the descriptive statistics and, where relevant, secondary inferential results, in a structured format for easy reference.

Table S9. Descriptive statistics for Experiment 1 across stimulus and pair types. **Mean / SD / Median / IQR / 95% CI:** participant-wise mean, standard deviation, median, interquartile range, and 95% confidence interval of the dependent variable; **N:** number of participants included.

Stimulus	Pair	Mean Acc	SD Acc	Median Acc	IQR Acc	95% CI Acc	Mean RT	SD RT	Median RT	IQR RT	95% CI RT	Mean Diff	SD Diff	Median Diff	IQR Diff	95% CI Diff	N
rectangle	CD	0.83	0.16	0.91	0.19	[0.79, 0.87]	4353.23	2324.47	3820.75	2813.28	[3769.90, 4936.56]	3.94	0.80	4.00	1.00	[3.74, 4.14]	61
rectangle	TD	0.92	0.12	1.00	0.08	[0.89, 0.95]	3541.06	1883.37	2963.75	1552.67	[3068.42, 4013.69]	3.44	0.83	3.50	1.10	[3.23, 3.65]	61
star	CD	0.70	0.19	0.67	0.27	[0.66, 0.75]	4828.74	2517.94	4477.50	3710.03	[4196.86, 5460.62]	3.91	0.96	3.83	1.47	[3.67, 4.15]	61
star	TD	0.88	0.16	1.00	0.18	[0.84, 0.92]	4641.23	2374.49	4357.25	2887.25	[4045.35, 5237.12]	3.73	0.80	3.91	1.20	[3.53, 3.93]	61

Table S10. Descriptive statistics for Experiment 2 choice percentages and reaction times. Choice percentages are in %, RT is in milliseconds. **CI95:** 95% confidence interval of the mean.

Alternative	Mean	SD	Median	IQR	CI95
Target	51.78	29.46	53.85	48.61	[51.30, 52.26]
Competitor	45.49	30.32	41.18	51.47	[44.99, 45.98]
Decoy	2.73	6.28	0	2.78	[2.63, 2.84]
RT	3942.83	3836.59	2369	3505.5	[3854.53, 4031.13]

Table S11. Descriptive statistics for Experiment 3. Choice percentages are reported in %, reaction times (**RT**) in milliseconds.**CI95:** 95% confidence interval of the mean.

Alternative	Mean	SD	Median	IQR	CI95
Target (%)	47.62	49.95	0.0	100.0	[46.62, 48.61]
Competitor (%)	48.34	49.98	0.0	100.0	[47.35, 49.34]
Decoy (%)	4.04	19.69	0.0	0.0	[3.65, 4.43]
RT (ms)	2326.76	2273.20	1596.0	1567.0	[2281.46, 2372.05]

Table S12. Experiment 4: Participant-wise descriptive statistics for Accuracy, RT, and Difficulty rating. Accuracy is proportion, RT is in milliseconds, Difficulty rating is on its scale.

Pair	Presentation	Accuracy_Mean	Accuracy_SD	Accuracy_Median	Accuracy_IQR	Accuracy_CI95	RT_Mean	RT_SD	RT_Median	RT_IQR	RT_CI95	Difficulty_Mean	Difficulty_SD	Difficulty_Median	Difficulty_IQR	Difficulty_CI95
CD	aligned	0.85	0.19	0.92	0.19	[0.79, 0.91]	3817.84	2098.70	3290.08	2111.18	[3163.84, 4471.84]	4.09	1.05	4.17	1.17	[3.76, 4.42]
CD	oblique	0.85	0.17	0.83	0.17	[0.80, 0.90]	4240.15	2086.01	3913.83	3105.68	[3590.10, 4890.19]	4.35	0.90	4.42	1.00	[4.07, 4.63]
TD	aligned	0.89	0.21	1.00	0.17	[0.82, 0.95]	2966.74	1527.09	2488.58	1232.88	[2490.86, 3442.61]	3.38	0.96	3.27	1.17	[3.08, 3.68]
TD	oblique	0.85	0.18	0.83	0.17	[0.80, 0.91]	3736.91	1717.62	3627.02	2437.17	[3201.66, 4272.16]	3.94	0.96	3.92	1.29	[3.64, 4.24]

Table S13. Repeated-measures ANOVA results for Experiment 4. Accuracy and RT are reported side by side for each effect.

Effect	Accuracy			RT		
	F(df1, df2)	p	η_p^2	F(df1, df2)	p	η_p^2
pair	1.26(1, 41)	0.269	0.03	9.35(1, 41)	0.004	0.19
presentation	0.8(1, 41)	0.375	0.02	6.69(1, 41)	0.013	0.14
pair:presentation	0.48(1, 41)	0.492	0.01	0.76(1, 41)	0.387	0.02

Table S14. Planned pairwise post-hoc comparisons for Accuracy and RT in Experiment 4. p-values are Holm–Bonferroni corrected. Cohen's d_z and 95% CI are reported.

Contrast	DV	t	df	p_{raw}	p_{holm}	d_z	95% CI
TD aligned vs CD aligned	Accuracy	1.13	41	0.266	0.882	0.17	[-0.13, 0.48]
TD oblique vs CD oblique	Accuracy	0.21	41	0.833	1.000	0.03	[-0.27, 0.34]
TD aligned vs CD oblique	Accuracy	1.24	41	0.220	0.882	0.19	[-0.11, 0.50]
CD aligned vs TD oblique	Accuracy	0.06	41	0.956	1.000	0.01	[-0.29, 0.31]
TD aligned vs CD aligned	RT	-2.88	41	0.006	0.019	-0.44	[-0.76, -0.12]
TD oblique vs CD oblique	RT	-1.68	41	0.101	0.202	-0.26	[-0.56, 0.05]
TD aligned vs CD oblique	RT	-4.04	41	0.00023	0.001	-0.62	[-0.95, -0.29]
CD aligned vs TD oblique	RT	0.25	41	0.804	0.804	0.04	[-0.26, 0.34]

REFERENCES

Bates, D., Mächler, M., Bolker, B., and Walker, S. (2015). Fitting linear mixed-effects models using lme4. *Journal of statistical software* 67, 1–48

Farmer, G. D., Warren, P. A., El-Deredy, W., and Howes, A. (2017). The effect of expected value on attraction effect preference reversals. *Journal of Behavioral Decision Making* 30, 785–793

Spektor, M. S., Kellen, D., and Hotaling, J. M. (2018). When the good looks bad: An experimental exploration of the repulsion effect. *Psychological science* 29, 1309–1320. Publisher: Sage Publications Sage CA: Los Angeles, CA

Spektor, M. S., Kellen, D., and Klauer, K. C. (2022). The repulsion effect in preferential choice and its relation to perceptual choice. *Cognition* 225, 105164. Publisher: Elsevier

Trueblood, J. S., Brown, S. D., Heathcote, A., and Busemeyer, J. R. (2013). Not just for consumers: Context effects are fundamental to decision making. *Psychological science* 24, 901–908

Wedell, D. H. (1991). enDistinguishing among models of contextually induced preference reversals. *Journal of Experimental Psychology: Learning, Memory, and Cognition* doi:10.1037/0278-7393.17.4.767
